# Phylogenetic Relationships, Speciation, and Origin of *Armillaria* in the Northern Hemisphere: A Lesson Based on rRNA and Elongation Factor 1-Alpha

**DOI:** 10.3390/jof7121088

**Published:** 2021-12-17

**Authors:** Junmin Liang, Lorenzo Pecoraro, Lei Cai, Zhilin Yuan, Peng Zhao, Clement K. M. Tsui, Zhifeng Zhang

**Affiliations:** 1 State Key Laboratory of Mycology, Institute of Microbiology, Chinese Academy of Sciences, Beijing 100101, China; lorenzo.pecoraro@gmail.com (L.P.); cail@im.ac.cn (L.C.); zhaopeng@im.ac.cn (P.Z.); zhangzhif0371@163.com (Z.Z.); 2 School of Pharmaceutical Science and Technology, Tianjin University, Tianjin 300072, China; 3 Institute of Subtropical Forestry, Chinese Academy of Forestry, Hangzhou 310029, China; zhi_lin_yuan@163.com; 4 Division of Infectious Diseases, Faculty of Medicine, University of British Columbia, Vancouver, BC V6T 1Z3, Canada; ctsui@mail.ubc.ca; 5 Department of Pathology, Sidra Medicine, Doha 2713, Qatar; 6 Shenzhen Key Laboratory of Marine Microbiome Engineering, Institute for Advanced Study, Shenzhen University, Shenzhen 518060, China

**Keywords:** phylogeography, species delimitation, allopatric speciation, molecular clock, ancestral area reconstruction

## Abstract

*Armillaria* species have a global distribution and play various roles in the natural ecosystems, e.g., pathogens, decomposers, and mycorrhizal associates. However, their taxonomic boundaries, speciation processes, and origin are poorly understood. Here, we used a phylogenetic approach with 358 samplings from Europe, East Asia, and North America to delimit the species boundaries and to discern the evolutionary forces underpinning divergence and evolution. Three species delimitation methods indicated multiple unrecognized phylogenetic species, and biological species recognition did not reflect the natural evolutionary relationships within *Armillaria*; for instance, biological species of *A. mellea* and *D*. *tabescens* are divergent and cryptic species/lineages exist associated with their geographic distributions in Europe, North America, and East Asia. While the species-rich and divergent Gallica superclade might represent three phylogenetic species (PS I, PS II, and *A. nabsnona*) that undergo speciation. The PS II contained four lineages with cryptic diversity associated with the geographic distribution. The genus *Armillaria* likely originated from East Asia around 21.8 Mya in early Miocene when Boreotropical flora (56–33.9 Mya) and the Bering land bridge might have facilitated transcontinental dispersal of *Armillaria* species. The Gallica superclade arose at 9.1 Mya and the concurrent vicariance events of Bering Strait opening and the uplift of the northern Tibetan plateau might be important factors in driving the lineage divergence.

## 1. Introduction

Understanding the biogeographical pattern and origin of fungi is important, especially for those fungal plant pathogens that cause severe economic losses. However, studying fungal biogeography is challenging. This has been attributed to shortcomings in delimiting species based on morphological characteristics, poor knowledge of the phylogeny in most fungal groups, rare fossil records, and the long-distance dispersal ability of spores to overcome geographic barriers [1]. For example, it took over a century of efforts to figure out the origin and dispersal pattern of *Pyricularia oryzae*, the famous pathogen of rice blast, since it was first discovered in 1892 [2,3]. Benefiting from the development of DNA technology and molecular analysis methods, there has been a breakthrough of fungal biogeography in recent decades [4,5,6]. Based on molecular calibration, the current distribution patterns and ancestral areas of many fungal groups have been well explained by vicariance events [7,8,9,10,11].

*Armillaria* species are well known as root rot pathogens, infecting over 500 host plants in both natural forests and silvicultural systems [12]. Individuals of *Armillaria* can reach immense sizes via growing rhizomorphs from one host plant to the neighboring plants, resulting in the largest known terrestrial organism in the world [13]. As mycorrhiza, some *Armillaria* species are associated with *Gastrodia elata* (“Tianma” in Chinese) [14] and *Polyporus umbellatus* that are widely used as traditional medicines in Asian countries [15]. The first biogeography study of *Armillaria* was reported in 2011, in which a non-Holarctic origin for *Armillaria* was suggested based on a phylogeny constructed by ITS, 28S, and TEF-1α [16]. In contrast, a recent study with a more robust multi-loci (28S, TEF-1α, *rpb2, tub*, *gpd*, and *act*) phylogeny of *Armillaria* inferred that Eurasia was the most probable origin [17]. Nevertheless, Eurasia is the combined continents of Europe and Asia, spanning from Asia Pacific to Atlantic Europe, and it is necessary to reconstruct the ancestral location of *Armillaria*.

The taxonomy of *Armillaria* has raised lots of controversial discussion. Currently, the accepted generic name is *Armillaria* Fr. Staude (1857) and *A. mellea* (Vahl: Fr.) P. Kumm is the type species [18,19]. Recently, *Armillaria* was narrowed down to include only annulate species, and *Desarmillaria* was introduced to accommodate two exannulate species (*D. ectypa* and *D. tabescens*) [17]. By now, a total of 278 *Armillaria* names are recorded in Index Fungorum (http://www.indexfungorum.org/Names/Names.asp) (accessed on 6 November 2021), but the validity of these name is often in debate [19]. On the delimitation of *Armillaria* species, the Biological Species Recognition (BSR) approach has prevailed since Korhonen [20] first employed this approach to delimit the biological species in the European population. *Armillaria* in many regions are recorded in biological species but they are not formally described, such as multiple Chinese Biological Species (CBS), CBS C, CBS F, CBS H, CBS J, CBS L, CBS N, CBS P, and CBS O [21]. Despite these limitations and weaknesses (e.g., ambiguous results and post-zygotic isolation not considered) in the practical application of BSR [12], these biological species represent great species diversity and are invaluable to understand the phylogeny of *Armillaria*. However, these biological species were not considered in previous studies of *Armillaria* phylogeography [16,17]. The Phylogenetic Species Concept (PSR) was introduced by Nixon and Wheeler, which emphasized the monophyly of species [22]. By using genealogical concordance phylogenetic species recognition (GCPSR), a large number of cryptic species in *Fusarium* [23,24] and *Collectotrichum* [25] have been uncovered under PSR. Similarly, phylogenetic analyses of DNA sequence data have been used to identify *Armillaria* strains to the species level, but the phylogenetic relationships of many biological species have not been clearly resolved [26,27].

The *Armillaria* genus has a worldwide distribution. Species occurring naturally in the northern hemisphere and southern hemisphere are phylogenetically distinct [28]. In the northern hemisphere, some species have transcontinental distributions, e.g., *A. mellea*, *A. gallica*, *A. ostoyae*, and *D. tabescens* (previously defined as *A. tabescens*), reported from Europe, Asia, and North America, and the North American *D. tabescens* has been separated as *D. caespitosa* recently [29]; *A. borealis*, *A. cepistipes*, and *D. ectypa* (previously defined as *A. ectypa*) distributed in both Europe and East Asia but not reported from North America; and *A. nabsnona* and *A. sinapina* distributed in both North America and East Asia [12,28,30]. Whereas, some other species are restricted to specific continents, such as *A. gemina*, *A. altimontana*, *A. calvescens*, and *A. solidipes* were only discovered in North America [31,32] and *A. singula*, *A. jezoensis*, and seven Chinese biological species were only known from East Asia [21,33]. Furthermore, there has been growing use of molecular approaches for species delimitation, such as the generalized mixed Yule-coalescent (GMYC) model [34,35], Poisson tree processes (PTP) model [36], and Bayesian multispecies coalescent method (BPP) [37]. These methods have not yet been applied in the phylogeny of *Armillaria*. Studying the species delimitation and relationships in *Armillaria* in the northern hemisphere would be useful to understand the genetic variability, as well as inter- and intraspecific divergence. The molecular data could infer the ecological factors and evolutionary processes that shape the various distribution patterns.

In this study, we collected 358 *Armillaria* samples in the northern hemisphere with an extensive distribution range covering Europe, North America, and Asia. First, we investigate the species boundaries and relationship of *Armillaria* spp. in the northern hemisphere according to GCPSR, PTP and BPP methods. Second, we aimed to answer the questions on the speciation event and evolutionary processes in *Armillaria*, such as allopatry or lineage divergence, due to geological and vicariance events and whether strains from different geographic regions could represent distinct species and have evolved independently. Third, we performed molecular dating to reconstruct the ancestral area/geographic distribution of *Armillaria*.

## 2. Materials and Methods

### 2.1. Fungal Specimens and Isolates

The investigation included 121 specimens loaned from six herbaria and these specimens were collected from 17 countries locating in Europe, North American, and Asia. Due to the absence of or limited Asian samples in previous phylogenetic studies, 105 strains isolated from sporocarps or rhizomorphs, covering 7 of 9 reported *Armillaria* species and 7 of 8 unnamed biological species in East Asia, were intensively collected in this study. In addition, 132 sequences from related strains/samples were downloaded from NCBI to represent species reported in Europe and North America. Since *Desarmillaria* species are the close relatives to *Armillaria* and recognized as *Armillaria* biological species, two *Desarmillaria* species were also included in this investigation. A total of 358 samples of *Armillaria* or *Desarmillaria* and related strains were included in the phylogenetic analyses. The detailed information of all samples used in this study is listed in the Supplementary Materials, Table S1.

For fresh sporocarps, spore prints were made on the same day of collection, stored in a cryogenic box, and brought back to the lab for single-spore isolation following the protocol described by Zhao et al. 1999 [38]. The collected rhizomorphs were first dipped in 50% ethanol for 15–20 s, and then sterilized in 20% hydrogen peroxide (H_2_O_2_) for 25–40 s depending on the thickness of the rhizomorphs. Both single spores and sterilized rhizomorphs were cultured on malt extract agar medium (12 g/L malt extract and 15 g/L Bacto Agar) amended with 2 mg/L benomyl and 100 mg/L streptomycin. All plates were incubated in darkness at 25 °C for 7–14 days to obtain isolated mycelia or rhizomorphs for DNA extraction.

### 2.2. DNA Extraction, PCR Amplification, and Sequencing

Genomic DNA was extracted from fresh cultured mycelia or rhizomorphs and sporocarp gills of dry specimens, at the junction of the stipe and pileus where spores are most possibly located, using a modified CTAB method [39]. Primers ITS1F and ITS4B [40] were used to amplify ITS region. Primers P-1 [41] and O-1 [42] were used to obtain the IGS-1 region. The partial TEF-1α gene was amplified with primer pairs EF595F/EF1160R [43]. Given that non-specific PCR products were obtained from some specimens with EF595F/EF1160R, a new primer pair, TEF-F (5′-GGCATCGAGGAGAGTCTTG-3′) and TEF-R (5′-TATCTCCAAGGACGGGCAGA-3′), was designed based on the TEF-1α gene of *Armillaria*. PCR reaction was performed in a total volume of 25 μL containing 2.5 μL of 10 × PCR buffer, 0.03 μL of dNTP mixture (10mM), 1 μL of each primer (10 pmol/μL), 0.15 μL of Taq polymerase (5 U/μL), and 1 μL of DNA template (50 ng/μL). PCR reactions were conducted in a ProFlexTM PCR system (Applied Biosystems, Foster City, CA, USA) under the following reaction conditions: denaturation at 94 °C for 5 min, followed by 35 cycles of denaturation at 94 °C for 30 s, annealing at 52 °C (for ITS) and 56 °C (for IGS-1 and TEF-1α) for 40 s and elongation at 72 °C for 1 min, a final elongation at 72 °C for 5 min. The purified products were sequenced in both directions on an ABI–3730 XL DNA Analyzer (Applied Biosystems, California).

### 2.3. Nucleotide Alignment and Data Matrices

The consensus DNA sequences were assembled using Seqman program in DNAstar (http://www.dnastar.com/t-seqmanpro.aspx, accessed on 12 July 2018). Alignments for each gene were generated using MAFFT 7 [44] and manually optimized in BioEdit 7.0.5 [45]. The three gene fragments were concatenated using Mesquite 3.31 [46].

Five data matrices were prepared. Dataset I (ITS+IGS-1+TEF-1α) including 358 samples was used to explore the phylogenetic relationship of all specimens and strains. Dataset I was also divided to three single-locus alignments to delimit phylogenetic species based on GCPSR [47]. Dataset II was set up to perform species delimitation using PTP model [36] and species validation in BPP program [48]. To balance the samples in each clade and satisfy the requirements in BPP program, 93 samples were chosen from Dataset I according to two following criteria: i) the number of samples in each well-supported clade ranged from 3 to 8 and strains from various geographic sites were chosen as much as possible; ii) samples with any gene locus absent were not included. Data III (ITS+IGS-1+TEF-1α) contained 270 concatenated sequences of samples in Gallica superclade and was used to perform neighbor-net phylogenetic network. Dataset IV (ITS+TEF-1α) was used to calibrate the most recent common ancestor (tMRCA) of the *Armillaria* clade, which included an extensive sampling of non-*Armillaria* outgroups from GenBank (Table S2). Dataset V (ITS+IGS-1+TEF-1α) was set up to run the second time of calibration, which included 22 *Desarmillaria*/*Armillaria* strains representing all phylogenetic species recognized in this study. In addition, the combined tree of Dataset V generated in BEAST was also employed for ancestral area reconstruction.

### 2.4. Phylogenetic Analyses and Species Delimitation

For Datasets I and II, Bayesian inference (BI) and Maximum Likelihood (ML) were performed using MrBayes 3.2 [49] and RAxML v.8 [50], respectively. The optimal evolutionary model was determined in MrModeltest [51] using the Akaike Information Criterion (AIC) for each locus. The selected substitution models for three loci were as follows: K81uf + Proportion Invariant + Gamma (K81uf + I + G) for ITS, General Time Reversible + I + G (GTR + I + G) for IGS-1, and Symmetrical model (SYM) + I + G for TEF-1α. The BI tree topology and posterior probabilities (PPs) were determined by a Markov Chain Monte Carlo (MCMC) algorithm of four chains with a random seed. Trees were sampled every 1000 generations with a burn in of the first 25% of trees. The MCMC analysis lasted until the average standard deviation of the split frequencies came below 0.01. The ML analysis was performed using RAxML, with 1000 bootstraps replicates, under the GTR-GAMMA model [50].

To delimit the species boundaries of *Armillaria*, three methods were applied. First, species were recognized by GCPSR following two steps [52]: (i) clades were genealogically concordant if they were present in >50% of the gene trees, and genealogically non-discordant if they were strongly supported (ML ≥ 70%; PP ≥ 0.95) in a single gene tree and not contradicted at or above this level of support in any other single gene tree; (ii) genetic differentiation and exhaustive subdivision criteria were applied [52]. Genetic differentiation required that lineages were well-differentiated, preventing minor terminal lineages from being recognized as phylogenetic species. Exhaustive subdivision required that all individuals were classified into phylogenetic species and no individuals were left unclassified. This technique involved tracing from the tip of the tree, and collapsing all lineages that were not subtended by an independent evolutionary lineage.

The second method used the PTP model, which stimulate the speciation or branching events in terms of number of substitutions based on a rooted phylogenetic tree [36]. The analysis was conducted on the web server for PTP (https://species.h-its.org/ptp/, accessed on 20 October 2020) using the RAxML tree estimated by Dataset II. Finally, a validation method, BPP, the reversible-jump Markov Chain Monte Carlo (rjMCMC) method [37], was used to test whether the candidate species suggested by GCPSR and PTP are reliable. This method tests alternative speciation hypotheses by collapsing or expanding nodes of the species tree using reversible-jump MCMC sampling in a fixed user-specified species tree and implemented through the software BPP 3.1 [53]. The program was run twice following the parameters below, species delimitation = 1, speciestree = 1, speciesmodelprior = 1, finetune ɛ = 1, usedata = 1 and cleandata = 0. The rjMCMC analyses consisted of 105 samples with a burn-in of 20%. The speciation event is validated if *pp* ≥ 0.95. 

### 2.5. Pairwise Homoplasy Index Test

The concordance of gene genealogies can be used to evaluate the significance of gene flow between groups within an evolutionary time scale [54]. To determine the recombination level between every pair of clades in the Gallica superclade, a pairwise homoplasy index (PHI) test using the GCPSR recognized clade (based on Dataset II) was performed in SplitsTree v.4.14 [55]. A PHI value less than 0.05 indicated significant recombination.

### 2.6. Neighbor-net Network Analysis

To better present the phylogenetic relationships and geographical distribution of species in the Gallica superclade, all samples clustered in the Gallica superclade (Dataset III) were used to draw a neighbor-net phylogenetic network. The software SplitsTree v.4.14 [55] was applied using the default parameters with K2P-derived distances.

### 2.7. Molecular Dating Analysis

A two-step calibration procedure was used to estimate node ages using BEAST 2.4.5 [56]. We used BEAUTi to create XML files for Datasets IV. Two nodes were calibrated using fossil data: (i) the initial diversification of the marasmioid fungi (*Marasmius alliaceus* and *Mycena pura*) based on a 90 million-year-old (Ma) fossil *Archaeomarasmius legetti* [57]; (ii) the divergence between Hymenochaetaceae and Fomitopsidaceae based on a 125 Ma fossil, *Quatsinoporites cranhamii* [58]. For lognormal settings, the first calibration was logmean = 2.5, logstdev = 0.5, and offset = 90.0 and the second calibration was logmean = 2.0, logstdev = 0.5, and offset = 125.0. The concatenated nexus files of ITS and TEF-1α were created and uploaded into BEAUTi following the settings: GTR model, uncorrelated relaxed clock with lognormal rate distribution, and the birth-death prior set. Three independent runs of 107 MCMC steps with random starting seeds were carried out, with sampling at every 1000 generations, following a burn-in of the initial 10% cycles. The convergence of runs was checked using Tracer with an effective sample size (ESS) > 200. The maximum clade credibility (MCC) tree was summarized using TreeAnnotator [59] and visualized in FigTree v1.4.3 (http://tree.bio.ed.ac.uk/software/figtree/, accessed on 15 July 2019) to obtain the means and 95% higher posterior densities (HPDs). 

### 2.8. Ancestral Area Reconstruction

To understand the biogeography of *Armillaria*, an ancestral area reconstruction analysis was inferred using the Bayesian Binary MCMC (BBM) analysis [60] and Dispersal-Extinction-Cladogenesis (DEC) model [61] and as implemented in the software of RASP 3.1 [62]. Based on the tectonic history of Laurasia, the geographic distributions for *Armillaria* were delineated into three areas: eastern Asia (A), Europe (B), and North America (C). Each taxon in our dataset was assigned to an area based on its current known distribution range. For the DEC model, the dispersal probability between A and B was unconstrained and the dispersal probabilities of the other two pairs, A←→C and B←→C, were constrained to 0.1 as summarized by previous studies [63]. The BBM analysis was conducted by setting the generation to 10 million with a burn-in of 20% and other parameters used default options. 

## 3. Results

### 3.1. Phylogenetic Analyses and Species Delimitation

**Figure 1:**
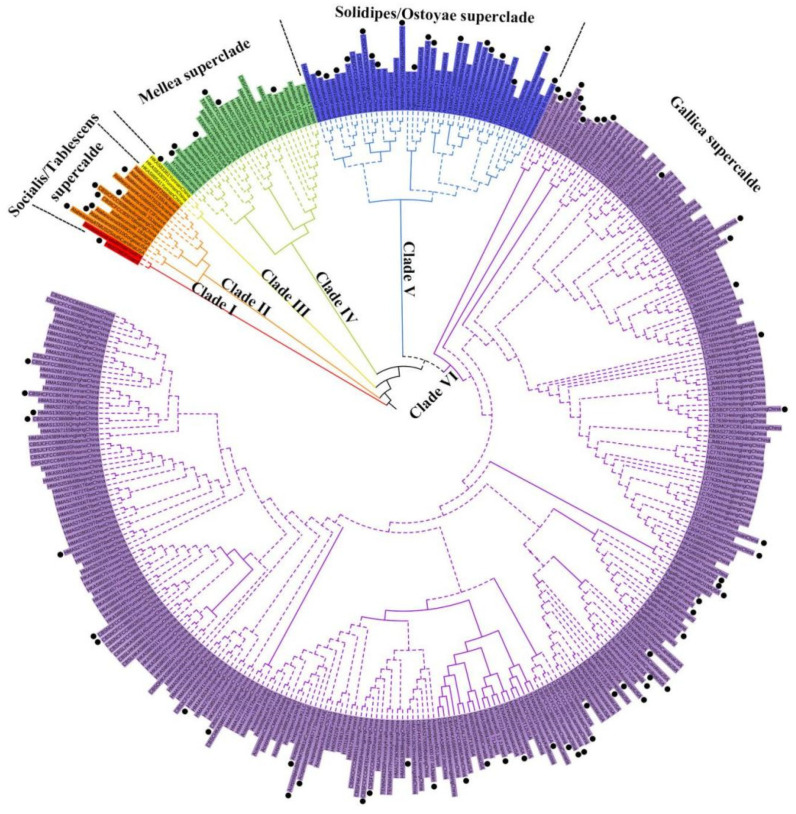
Phylogenetic tree of 358 *Armillaria* and *Desarmillaria* samples inferred from maximum likelihood and Bayesian analyses based on Dataset I (ITS+IGS-1+TEF-1α). Only bootstraps (LB) over 70% and Bayesian posterior probabilities (PPs) over 0.95 are shown on the branches. The samples labeled with black dots were chosen for the species delimitation analysis presented in Figure 2.

The concatenated sequences of Dataset I (ITS+IGS-1+TEF-1α) were 2010 bp in length (with gap), containing 821 polymorphic sites (320 from IGS-1, 280 from TEF-1α, and 221 from ITS). The nucleotide diversity (Pi) of TEF-1α and IGS-1 was 5.0 × 10^−2^ and 4.9 × 10^−2^, relatively higher than that of ITS (Pi = 1.6 × 10^−2^). The ML and BI analyses yielded identical tree topology with six well-supported clades. The concatenated tree inferred from ML analysis is shown in Figure 1 and the single gene trees are shown in Figure S1. According to the “superclade” identified by Klopfenstein et al. 2017 [27], 226 samples sequenced in our study were assigned to the Gallica superclade (185 samples), Solidipes/Ostoyae superclade (26 samples), Mellea superclade (12 samples), and *Desarmillaria* genus (3 samples). Three Chinese strains isolated from rhizomorph samples associated with *Gastrodia elata* in the natural field clustered in an additional clade, Clade III. Since *Desarmillaria* species do not produce rhizomorphs in nature, Clade III could represent a new superclade in *Armillaria*. Whether Clade III is the most ancient *Armillaria* clade needs more samples together with southern hemisphere species to verify. These strains were not included in the following species delimitation due to the absence of IGS-1 sequences. 

**Figure 2:**
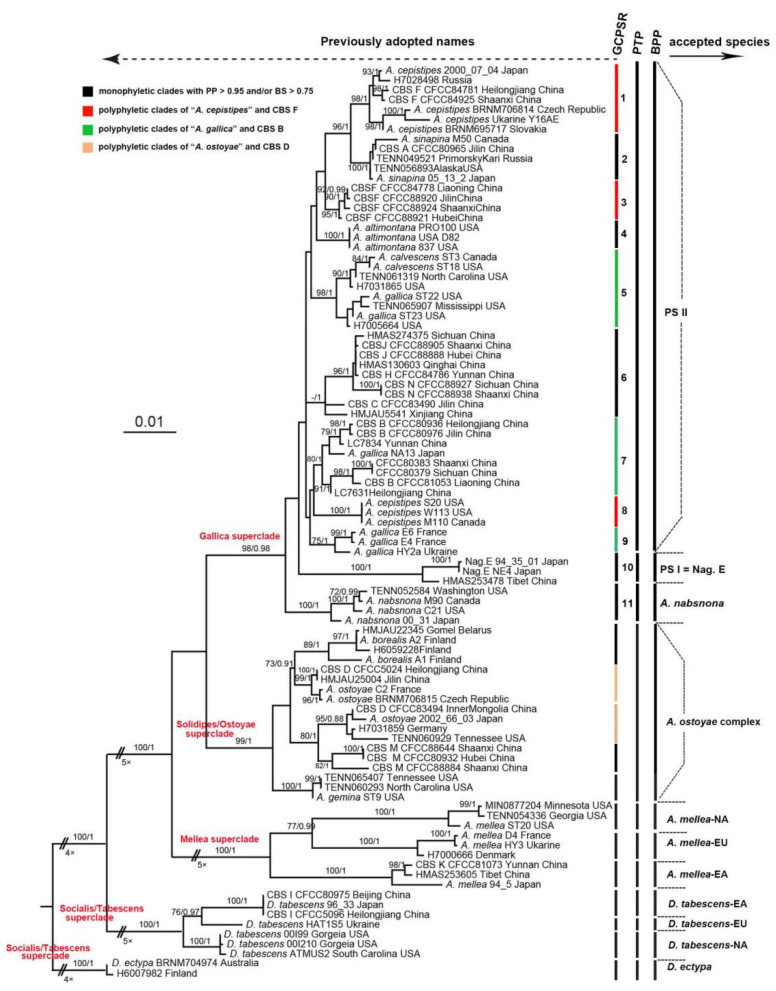
Species delimitation of *Armillaria* and *Desarmillaria* based on a phylogenetic constructed using Dataset II (ITS+IGS-1+TEF-1α)) consisting of 93 representative strains. Only maximum likelihood bootstraps (LBs) over 70% and Bayesian posterior probabilities (PPs) over 0.95 are shown on the branches.

For Dataset II (93 representative samples), all species delimitation methods yielded a consistent result that previously defined *D. tabescens* and *A. mellea*, where each consisted of three strong supported clades geographically limited to East Asia, North America, and Europe. Here, we recorded *D. tabescens* as *D. tabescens*-EA, *D. tabescens*-NA, and *D. tabescens-*EU. Similarly, *A. mellea* herein was recorded as *A. mellea*-EA, *A. mellea*-NA, and *A. mellea*-EU. In the Ostoyae/Solidipes superclade, due to limited samples, the phylogenetic relationships among representatives were not completely resolved. *Armillaria ostoyae* collected from Japan and European countries were clustered in two separate clades, suggesting that *A. ostoyae* was not monophyletic (Figure 2). Three CBS M (CFCC 88644, CFCC 80932, CFCC 8884) specimens, previously defined as *A. borealis* due to their mating compatibility test with European *A. borealis* [21], however, were not clustered together but clearly phylogenetically separated by two *A. ostoyae* clades (Figure 2). In the Gallica superclade, 11 candidate taxonomic units (according to 11 clades in Figure 2) were designated based on the bootstrap or posterior probability values according to GCPSR. Whereas, PTP analysis only recognized three species, i.e., *A. nabsnona* (clade 11), Phylogenetic species I (PS I, i.e., clade 10, previously defined as Nag. E) [64], and PS II (clade I to clade 9) (Figure 2 and Figure S2).

In order to test the discrepancy of the hypothesized species inferred from PTP and GCPSR, additional analysis was performed by running BPP. A dataset was composed of strains of three potential species (*A. nabsnona*, PS I, and PS II) and three reference species, three cryptic species in the Mellea superclade (*A. mellea*-EU, *A. mellea*-EA, and *A. mellea*-NA) [28]. Four combinations with a varied population size and divergence rates were analyzed. Both analyses with large ancestral population size (G*θs*: 1, 10) supported four reference species and three species of the Gallica superclade delimited in PTP based on the GCPSR result; clade 10 and clade 11, corresponding to *A.* *nabsnona* and PS I, were still strongly supported while several potential species in clade 1 to clade 9 were not supported to be distinct species due to low posterior possibility values (<0.95) (Table 1). Both PTP and BPP supported that the Gallica superclade contained three phylogenetic species. Previously defined “*A. nabsnona*” and “Nag. E” were confirmed to be two well-supported species and recorded as *A. nabsnona* and PS I in this study while previously defined “*A. galli*ca”, “*A. cepistipes*”, “*A. calvescens*”, “*A. altimontana*”, and “*A. sinapina*” and seven Chinese Biological Species” (CBS C, CBS F, CBS H, CBS J, CBS L, CBS N, and CBS O) were collectively recognized as a single species and recorded herein as PS II. 

When applying PHI tests, no significant recombination was detected between pairs of *A. nabsnona*, PS I, and PS II, but significant recombination was detected among different clade-pairs within PS II (Table 2), which indicated that the reproduction isolation had formed among *A. nabsnona*, PS I, and PS II but not formed completely within PS II.

### 3.2. Phylogenetic Relationships in PS II

**Figure 3:**
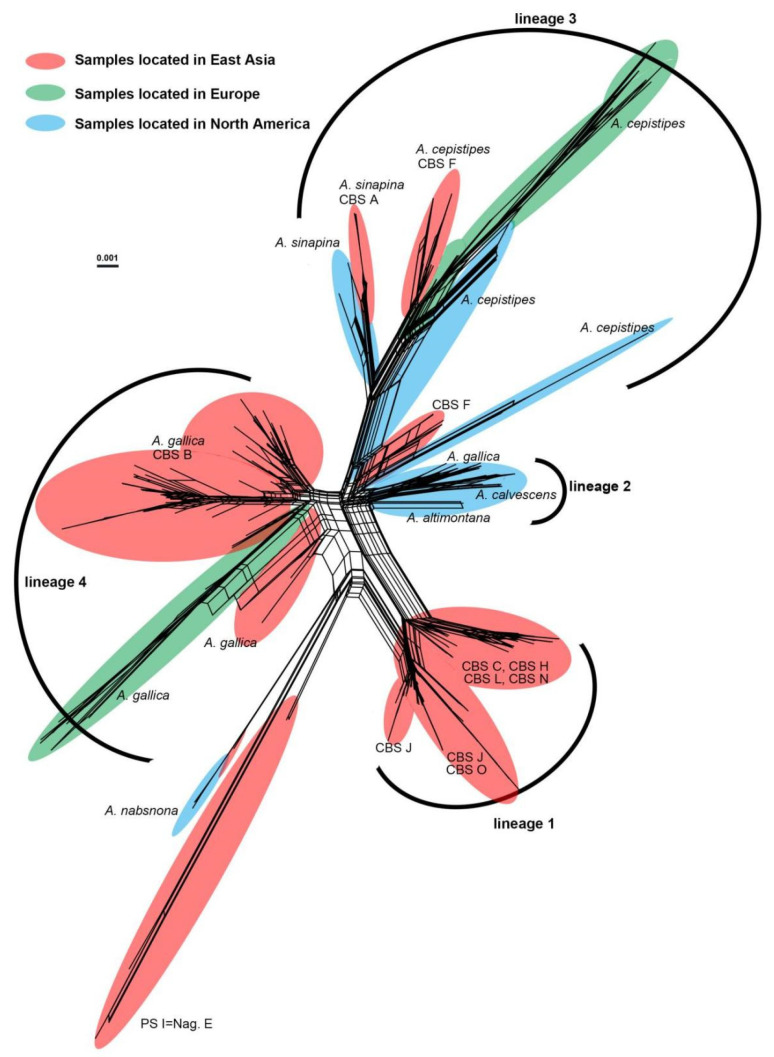
A neighbor-net phylogenetic network based on partial sequences of ITS, IGS-1, and TEF-1α from 270 *Armillaria* samples in the Gallica superclade created by SplitsTree v4.14.4 with K2P distance. The names of previously defined species were listed nearby the lineages. Samples located in East Asia, Europe, and North America were highlighted in red, green, and blue, respectively. Detailed information of 270 *Armillaria* samples is described in Table S1.

The neighbor-net network revealed the presence of divergent lineages in the Gallica superclade. The Gallica superclade was divided into multiple lineages as *A. nabosnoa* and PS I (Nag. E) were recognized as distinct distant lineages (Figure 3). Within PSII, previously unnamed six CBS “species” (CBS C, CBS H, CBS J, CBS L, CBS N, and CBS O), together with many Chinese samples were grouped as lineage 1. Among the 87 samples in this lineage, 81 (93%) samples were collected from Qinghai-Tibet Plateau and adjoining areas covering Tibet, Qinghai, Xinjiang, Sichuan, Yunnan, Shaanxi, and Guizhou Provinces (Table S1). Therefore, we recorded this lineage as the Chinese endemic lineage. Lineage 2 contained North America-centric samples, including two North America-specific species, *A. altimontana* and *A. calvescens,* as well as the specimens of *A. gallica* in North America. Lineage 3 comprised CBS A, CBS F, *A. cepistipes*, and *A. sinapina*. The divergence within *A. cepistipes* and *A. sinapina* could indicate the existence of geographical divergence. *Armillaria sinapina* and CBS A were clustered together, but samples from North America and East Asia formed two sister clades. *A. cepistipes* collected from North America were clustered in two distinct clades and CBS F were grouped to two distinct clades, one with *A. cepistipes* from Europe and North America and another with only Chinese samples. In lineage 4, *A. gallica* collected from East Asia and Europe as well as CBS B were included.

### 3.3. Estimation of Divergence Time

**Figure 4:**
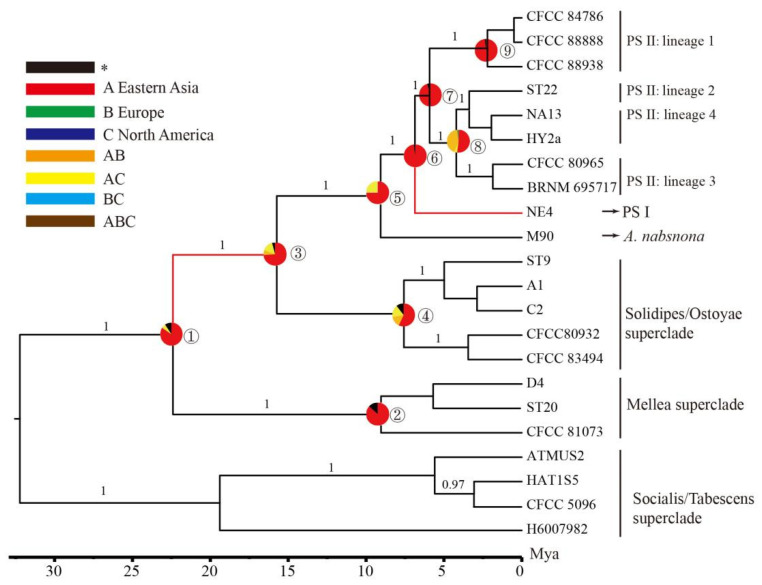
Chronogram of *Armillaria* from the northern hemisphere. The time-scale is set to the mean divergence dates produced in BEAST. The most recent common ancestor (tMRCA) of *Armillaria* and *Desarmillaria* (node 1) was estimated based on Dataset IV and its chronogram is shown in Figure S3. Numbered nodes refer to mean divergence dates, with their 95% HPD and ancestral state provided in Table 3. The pie chart in each node indicates the possible ancestral distributions inferred from Bayesian Binary MCMC analysis (BBM) implemented in RASP. Red branches identify dispersal events inferred by RASP based on the Dispersal-Extinction-Cladogenesis (DEC) model.

The first molecular dating with two fossils suggested that the most recent common ancestor (tMRCA) of *Armillaria* and *Desarmillaria* was estimated at early Miocene (21.8 Mya, 95% HPD: 13.1–32.6 Mya, Figure 4). The second calibration showed the divergence date of the Solidipes/Ostoyae superclade and Gallica superclade was estimated at 15.7 Mya (95% HPD: 3.5–36.2 Mya) (node 3 in Table 3, Figure 4). The diversification of the Gallica superclade was estimated to have occurred at 9.1 Mya (95% HPD 21.4–1.2 Mya) (node 5 in Table 3, Figure 4). The PS I separated from its tMRCA during late Miocene to Pleistocene (6.9 Mya, 95% HPD:1.1–16.2 Mya) (node 6 in Table 3, Figure 4). Four lineages of PS II diverged during late Miocene to Pleistocene (95% HPD: 10.1–0.6 Mya) (node 8 in Table 3, Figure 4).

### 3.4. Ancestral Areas of Armillaria

The most probable ancestral areas for internal nodes of *Armillaria* are not completely consistent in the BBM and DEC analyses. In the BBM analyses, most nodes were inferred as a single region of origin, such as East Asia or North America, and East Asia was the most probable ancestral area for *Armillaria*. While in the DEC analyses, multi-regional origins covering East Asia, Europe, and North America were inferred in several nodes (Table 3), but the relative probabilities of the ancestral area for some nodes are very low (nodes 1 and 3). Some nodes were consistently supported by BBM and DEC analyses, such as the ancestral areas of *A. nabosnona* (node 5), PS I+PS II (node 6), PS II (node 7), and lineage 1 in PS II, which were all suggested to originate from East Asia.

## 4. Discussion

Based on phylogenetic analyses and multiple species delimitation methods, our results supported the monophyly of “biological species” (“BS”) *D. tabescens* and *A. mellea,* but within each of these two “BS”, allopatric speciation has been detected. For instance, “BS” *A. mellea* was composed of three distinct phylogenetic species geographically limited to North America (*A. mellea*-NA), East Asia (*A. mellea*-EA), and Europe (*A. mellea*-EU) (Figure 2). “BS” *D. tabescens* also presented the same geographic grouping (NA, EA, and EU). Whereas in the Gallica superclade, 11 “biological species” were revealed to represent only 3 phylogenetic species (Figure 2, Table 1), i.e., the previously recognized CBS C, CBS H, CBS J, CBS L, CBS N, and CBS O [21], although clustered together but failed to be distinguished from each other. In the Solidipes/Ostoyae superclade, CBS D and “BS” *A. ostoyae* mixed together but distributed in two non-adjacent phylogenetic clades, with each clade consisting of both CBS D and “BS” *A. ostoyae*. This interlaced distribution was also shown in a previous study [26]. Therefore, the previously established relation between “BS” *A. ostoyae* and CBS D should be challenged [69], because “BS” *A. ostoyae* is not monophyletic, dividing into at least two distinct lineages (Figure 2). The pronounced discrepancies between “biological species” and phylogenetic grouping suggested that biological species recognition did not reflect the natural evolutionary relationships within *Armillaria*. Besides its ambiguous criterion and complicated operability as criticized by many mycologists [70], biological recognition is particularly inadequate for *Armillaria* species. In previous studies, the sole detection of hyphal fusion and diploidy in “compatibility tests” was improperly considered as the evidence of mating success in *Armillaria* [20]. Additionally, hyphal fusion can occur at the interspecific level, as reported in smut fungi from different families [70]. Mating success requires not only hyphal fusion but also fertile offspring production to be achieved. Previous compatibility tests did not check whether the fused diploid could produce fertile offspring. A similar example is *Neurospora tetrasperma* complex [71]. Nine phylogenetic species were recognized following PSR. If using the traditional broad biological species recognition, hyphal fusion, these nine phylogenetic species constituted a single biological species. However, when examining the reproductive success, such as the viability and fertility of offspring, BSR also supports the nine species delimitation. Nevertheless, it is difficult to measure the fertility of offspring in macrofungi, e.g., *Desarmillaria* and *Armilllaria*. So far, the in vitro fruiting system of *Armillaria* was only reported in the “BS” *A. ostoyae* and *A. mellea*-NA lineage [72,73], while hyphae-fused *Armillaria* “biological species” have been widely and mistakenly recognized. Speciation is a dynamic and ongoing evolutionary process, through which a species arises. The inconsistency among species concepts could be caused by different phenotypic and/or genotypic features used in different species concepts/recognition [74]. With more and more genome data becoming available, genome sequence-based classification and identification has appealed recently [74,75]. The validity of multi-geographic limited species in Tabescens and Mellea superclades should be evaluated with genome data in the future. Our estimates of the tMRCA of *Armillaria* in the northern hemisphere suggest it arose at 21.8 (32.6–13.1, 95% HPD) Mya, overlapped with a previous estimate (43.4–17.7 Mya, 95% HPD) based on a global phylogeny of *Armillaria* [17], even though a different set of loci was used. During this time period, Boreotropical flora (56–33.9 Mya) [76] was establishing (if adopting Koch’s estimation) or had resulted in a global shift of vegetation in the northern hemisphere. Many large and fast-growing trees, such as alpine coniferous deciduous forests, had emerged [77]. These plants were frequently recorded as common substrates of *Armillaria* spp. [21,66,78], which may facilitate long-distance transcontinental dispersal.

In our case, allopatric speciation due to geographic isolation was shown in “BS” *A. mellea* and *D. tabescens*. Three clades corresponding to their geographic locations in Asia, Europe, and North America were revealed in *A. mellea*. These geographic clades were well supported and recognized as distinct species based on data analysis (Figure 2). The North America clade can be further divided into eastern and western clades based on microsatellite loci [79]. These geographic clades were accepted as cryptic species in the Mellea superclade recently [26]. Besides, *A. mellea* was also reported from Africa [80], Mexico [81], and Iran [82]. A more comprehensive phylogeny with samples from different countries should be constructed to test the existence of cryptic species in the Mellea superclade. Similarly, three continental-specific clades of *D. tabescens* were recognized as distinct species and highly supported by the molecular delimitation methods. The DNA variation among three continental specific clades of *D. tabescens* is even greater than the ever proposed species in the Gallica superclade. Strains from East Asia, Europe, and North America were considered as conspecific based on incongruent placement on two single gene trees (IGS-1 and TEF-1α) [26]. Nevertheless, a recent five gene phylogeny (28S, TEF-1α, *rpb2*, *act*, and *gpd*) supported the phylogenetic variation between *D. tabescens*-NA and *D. tabescens* from Eurasia and introduced *D. tabescens*-NA as a new species, *D. caespitosa*, due to significant morphological differences including wider basidiospores, narrower cheilocystidia, and caulocystidia [29]. Our results suggested that not only *D. tabescens*-NA, but also *D. tabescens-*EA could present a new species. Our results supported a recent speculation that even more crytic species existed in the Tabescens superclade estimated by ITS1 or ITS2 from public databases [83]. Previous studies indicated several factors could contribute to allopatric speciation, such as genetic drift in spatial populations and natural selection [84,85]. With more available *Armillaria* genome sequences [86], the mechanisms of allopatric speciation and adaptations in Mellea and Tabescens superclades should be analyzed at the genomic level in future study.

The first species separated from the crown node of the Gallica superclade was *A. nabsnona*, which showed a disjunctive distribution in East Asia and the west coast of North America [30,87]. The divergence time of *A. nabsnona* was around 9.1 Mya, earlier than the opening of the Bering Strait (node 5: Table 3 and Figure 4). The long-distance transcontinental dispersal, incremental dispersal over land, and vicariance may have contributed to the disjunctive distribution of *A. nabsnona*. With new sampling in this study, PS I, previously recorded as Nag. E only reported from Japan, showed a broader distribution including one specimen from Tibet, China (Figure 2, Table S1).

In the Gallica superclade, multiple delimitation methods recognized several previously defined “biological species” as a single phylogenetic species, PS II (Figure 2), regardless of their variability in host range and morphologies. However, the phylogenetic network indicated lineage divergence in PS II, which is experiencing an ongoing speciation process. *Armillaia* samples from Qing-Tibet Plateau formed a distinct lineage (lineage 1) and molecular dating showed that this lineage arose at 2.2 Mya (6.2–0.1 Mya, 95% HPD) (Figure 4), in concurrence with Pliocene uplift of the Northern Tibetan Plateau (since 4.5 Mya) [88]. We hypothesized that the appearance of this endemic *Armillaria* lineage was driven by the uplift of the Northern Tibetan Plateau. It has been well documented that the Pliocene uplift of Himalaya resulted in critical local climate change at around 5 Mya [89,90]. High species diversity and potential origins of many well-known species in different groups from the Himalaya areas have also been reported, such as *Saccharomyces cerevisiae* in fungi [91], *Quercus* spp. in plants [92], and *Coelotine* spp. spiders in animals [93]. Therefore, the divergence of lineage 1 could be a consequence of adaptive evolution and geographic isolation. Neither recombination tests of the Parsimony Tree Length Permutation Test (PTLPT) nor linkage disequilibrium (*r*d) showed signal of recombination within lineage 1 (Figure S4), suggesting that lineage 1 is expanding its population size via the clonal reproductive mode. Several commercial strains (HKAS 86556, HKAS 86,557, and HKAS 86543), widely used in the growth of *Gastrodia elata* (a traditional medicinal plant), clustered in lineage 1. Frequent introduction of these commercial plants may have contributed to its clonal expansion.

Lineage 3 and lineage 4 comprised clades of different geographic origins (Figure 3). The tMRCA (node 9) of lineage 2, lineage 3, and lineage 4 was estimated at 4.2 Mya (Figure 4, Table 3), later than the opening of the Bering Strait (5.5–5.4 Mya) [94]. Therefore, the vicariance event, such as the opening of the Bering Strait, could have driven the divergence of lineages located in North America and East Asia. In lineage 3, samples located in North America and East Asia were genetically separated (Figure 3) and a similar genetic distinction was also revealed in other wood-decay fungi, i.e., *Coniferiporia sulphurascens* [95]. Although *Armillaria* could disperse over long distances via basidiospores [16], it is limited by the Pacific Ocean [96]. One possible explanation was that the common ancestors of continental samples in lineage 3 spread via the Bering land bridge to North America to attain a transcontinental distribution and subsequently diverged when vicariance events occurred.

A previous study roughly suggested Eurasia was the most probable ancestral area for *Armillaria* [17]. In this study, with much more extensive samplings in the northern hemisphere, the source of *Armillaria* origin was narrowed to East Asia with 88% probability in BBM analysis (Figure 4 and Table 3). Although the DEC analysis suggested that the *Armillaria* genus was probably a multi-range origin, the probability was too low (ABC, 33%, Table 3) to be accepted. The most ancient clade in *Armillaria* is Clade III (Figure 1), which included three samples representing an undescribed phylogenetic species so far known only from China. The earliest lineage diverged from Clade III is *A. mellea*-EA, an East Asian geographically limited species, which included both heterothallic and homothallic reproductive systems [12]. The phylogeny and neighbor-net analysis of northern *Armillaria* spp. revealed that samples from East Asia distributed in every major evolutionary clade or lineage, indicating a diversity center with high species richness and genetic diversity of *Armillaria* in East Asia. As estimated by Koch et al. [83], Eastern Asia represents the biogeographic region with the highest species richness. In a detailed analysis of the largest evolutionary clade, the Gallica superclade, both BBM and DEC analyses supported an East Asian origin (Figure 4 and Table 3). Therefore, it is reasonable to speculate that East Asia is the origin of the *Armillaria* genus in the northern hemisphere, as well as the origin of most major evolutionary lineages in *Armillaria*.

## 5. Conclusions

In summary, the phylogenetic species approach based on rRNA and TEF-1α indicated the presence of cryptic species and lineages within *Armillaria*, giving more natural species delimitation than biological species recognition. Geographic isolation has been implicated to be a key determinant in the speciation and lineage divergence. *Armillaria* was inferred to originate from East Asia at Early Miocene and the basal clade had evolved to three distinct species after dispersing to other continents. However, in the Gallica clade, most previously defined species could represent lineages of PS II, a super species experiencing an ongoing speciation process. Divergence time estimation suggested that both the transcontinental dispersal within the boreal floristic kingdom and vicariance events during early Pliocene could have led to a disjunctive distribution and lineage divergence of *Armillaria* species in the Gallica superclade.

## Figures and Tables

**Table 1:** Results from BPP analyses for the Gallica superclade assuming 3-species or 11-species models.

Priors	Posterior Probability	Posterior Probability for Delimited Species														
		*A. mella* –EA	*A. mella* –NA	*A. mella* –EA	*A. mella* –EU	PS I /Clade 10	PS II	Clade 9	Clade 8	Clade 7	Clade 6	Clade 5	Clade 4	Clade 3	Clade 2	Clade 1
*θ ~* G(1, 10), *τ*_0_ ~ G(1, 10)	P[3] = 1.000	1.000	1.000	1.000	1.000	1.000	1.000	–	–	–	–	–	–	–	–	–
*θ ~* G(1, 10), *τ*_0_ ~ G(2, 1000)	P[3] = 1.000	1.000	1.000	1.000	1.000	1.000	1.000	–	–	–	–	–	–	–	–	–
*θ ~* G(2, 1000), *τ*_0_ ~ G(1, 10)	P[3] = 1.000	1.000	1.000	1.000	1.000	1.000	1.000	–	–	–	–	–	–	–	–	–
*θ ~* G(2, 1000), *τ*_0_ ~ G(2, 1000)	P[3] = 1.000	1.000	1.000	1.000	1.000	1.000	1.000	–	–	–	––	–	–	–	–	–
*θ ~* G(1, 10), *τ*_0_ ~ G(1, 10)	P[11] = 0.215	1.000	1.000	1.000	1.000	1.000	–	0.691	1.000	0.694	0.990	1.000	0.998	0.497	0.335	0.780
*θ ~* G(1, 10), *τ*_0_ ~ G(2, 1000)	P[11] = 0.373	1.000	1.000	1.000	1.000	1.000	–	0.961	1.000	0.961	0.783	1.000	1.000	0.951	0.595	0.427

**Table 2:** PHI test (above diagonal) and compatible test (below diagonal) of phylogenetic clades within the Gallica superclade.

Species	PS II ^a^									PS I	*A. nabsnona*
	Clade 1 ^b^	Clade 2	Clade 3	Clade 4	Clade 5	Clade 6	Clade 7	Clade 8	Clade 9	Clade 10	Clade 11
	*A. Cepistipes* EA&EU ^c^	*A. sinapina*	CBS F	*A. altimontana*	*A. calvescens*	CBS C, CBS J, CBS L, CBS H, CBS N	*A. gallica* EA	*A. cepistipe* NA	*A. gallica* EU	Nag. E	*A. nabsnona*
Clade 1		0.03	0.94	0.7	0.305	0.001	0.138	0.18	0.048	0.091	0.067
Clade 2			0.30	0.35	0.107	0.001	0.027	0.37	0.219	0.085	0.277
Clade 3				1	0.39	0.003	0.306	1	1	0.092	0.159
Clade 4					0.065	0.031	0.322	1	1	0.091	0.329
Clade 5						0.021	0.089	0.15	0.15	0.051	0.269
Clade 6							0.008	0.001	0.001	0.108	0.037
Clade 7								0.001	0.001	0.254	0.081
Clade 8									1	0.096	0.12
Clade 9										0.237	0.156
Clade 10											0.089
Clade 11											

^a^ Three *Armillaria* phylogenetic species delimited by PTP and BPP in Gallica superclade ^b^ Eleven phylogenetic clades supported by GCPSR in Gallica superclade (Figure 2). ^c^ Previously defined morphological and biological species in Gallica superclade. EA = East Asia, EU = Europe, and NA = North America. Note: The values in above diagonal are recombinational possibility calculated by PHI test. The below diagonal are hyphal fusion events reported in previous studies [21,65,66,67,68]. Blue cells represent significant recombination with *p* < 0.05 or compatible results in previous mating tests and red cells represent nonsignificant recombination with *p* > 0.05 in PHI test or incompatible mating interactions reported previously.

**Table 3:** Divergence time estimates of BEAST analyses for internal nodes of northern hemisphere *Armillaria*, with results of ancestral range estimation using the BBM and DEC models.

Node	Species/Lineage	Mean Divergence Time (95% HPD Mya)	Ancestral Area Reconstruction (Area/Relative Probability)	
			BBM *	DEC *
1	*Armillaria*	21.8 (13.1–32.6)	A/0.84	ABC/0.3
2	*Mellea* superclade	9.0 (1.5–21.6)	A/0.87	ABC/1
3	Solidipes/Ostoyae superclade+*A. nabsnona*+PS I+PS II	15.7 (3.5–36.2)	A/0.74	ABC/0.48
4	Solidipes/Ostoyae superclade	7.6 (0.8–18.1)	A/0.57	ABC/1
5	Gallica superclade	9.1 (1.2–21.4)	A/0.73	A/0.71
6	PS I+PS II	6.9 (1.1–16.2)	A/0.98	A/1
7	PS II	5.9 (0.8–14.0)	A/0.95	A/0.74
8	PS II: lineage 2, lineage 3 and lineage 4	4.2 (0.6–10.1)	A/0.51	ABC/1
9	PS II: lineage 1	2.2 (0.1–6.2)	A/0.99	A/0.84

* BBM = Bayesian Binary Markov chain Monte Carlo model [60], DEC = Dispersal-Extinction-Cladogenesis model [61]. Both BBM and DEC analyses were implemented in RASP 3.1 [62].

## Data Availability

Data of this study are included in the article or Supplementary Materials.

## Supplementary Materials

The following are available online at https://www.mdpi.com/article/10.3390/jof7121088/s1, Figure S1: Single locus phylogenetic analyses of 358 *Desarmillaria* and *Armillaria* strains used in this study based on ITS (a), IGS-1 (b) and TEF-1α (c). Only maximum likelihood bootstraps (LB) over 70% and Bayesian posterior probabilities (PP) over 0.95 were showed on the branches, Figure S2: Results of the PTP analysis based on the BI and ML topologies. Putative species clusters are indicated using transitions between blue and red branches. Figure S3: Molecular dating of tMRCA of *Armillaria* and *Desarmillaria* with 27 reference strains representing major clades of Agaricomycetes, Dacrymycetes and Tremellomycetes. The time-scale is set to the mean divergence dates produced in BEAST v. 2.4.5, Figure S4: Recombination test of *Armillaria* Chinese specific population of PS II based on linkage disequilibrium (rd) and parsimony tree lengths (PTLPT). Ranges of PTLPT and rd were conducted from 1000 randomized permutations using Multilocus v. 1.3, Table S1: Detailed information of 358 *Armillaria* and *Desarmillaria* samples used in this study, Table S2: Species and their sequences accession number used for molecular clock analysis.
