# Supplementary material for: Phylogenetic Relationships, Speciation, and Origin of Armillaria in the Northern Hemisphere: A Lesson Based on rRNA and Elongation Factor 1-Alpha
Source: J Fungi (Basel). 2021 Dec 17;7(12):1088. doi: 10.3390/jof7121088 (PMC8705980; doi:10.3390/jof7121088)
Supplement: Supplementary file 1 [file jof-07-01088-s001.zip › supplementary files/Table S1 strains information.pdf]

Table S1 Information of all samples used in this study.

Phylogenetic species		Biological species	Isolate ID	Origin	Location	Substance	GenBank accession numbers			References
Species	lineage*						ITS	IGS-1	TEF-1a	
Desarmillaria. ectypa		*A. ectypa*	BRNM 704974	strain	Australia	On soil, among Sphagnum	EU257720	EU257712	EU251403	Antonin et al. 2009
D. ectypa		/	H6007982	sporocarp	Finland	grass herb forest	MG931757	MG931531	OL742434	this study
D. tabescens-EA		*A. tabescens*	96 1 8	strain	Japan	Unknown	AB510867	AB510823	AB510804	Hasegawa et al.
D. tabescens-EA		*A. tabescens*	96_3_3	strain	Japan	Unknown	AB510868	AB510824	AB510805	Hasegawa et al.
D. tabescens-EA		CBS I[2]	CFCC 5906	single spore	Heilongjiang, China	Unknown	MG931732	MG931506	OL742427	this study
D. tabescens-EA		CBS I[1]	CFCC 80975	single spore	Beijing, China	Conifer and broadleaf forest	MG931733	MG931507	OL742426	this study
D. tabescens-EU		*A. tabescens*	HAT1S5	strain	Ukraine	Unknown	HQ232292	HQ232284	HQ285906	Coetzee et al. 2015
D. tabescens-EU		*A. tabescens*	HAT2S5	strain	Ukraine	Unknown	HQ232293	HQ232285	HQ285907	Coetzee et al. 2015
D. tabescens-EU		*A. tabescens*	HAT5S3	strain	Ukraine	Unknown	HQ232294	HQ232286	HQ285908	Coetzee et al. 2015
D. tabescens-EU		*A. tabescens*	HKAS 86605	tester strain	Slovenia	Unknown	KT822337	—	KT822439	Guo et al. 2016
D. tabescens-EU		*A. tabescens*	ET3	strain	France	Unknown	—	JF288740	JF746929	Mulholland et al.
D. tabescens-NA		*A. tabescens*	ATMUS2	stripe	South Carolina, USA	Unknown	AY213588	AY509189	JF313113	Kim et al. 2006
D. tabescens-NA		*A. tabescens*	OOI210	sporocarp	Georgia, USA	Unknown	AY213589	AY509191	JF313111	Kim et al. 2006
D. tabescens-NA		*A. tabescens*	OOI99	sporocarp	Georgia, USA	Unknown	AY213590	AY509192	JF313112	Kim et al. 2006
A. mellea-EA		CBS G[1]	HKAS 86592	strain	Guizhou, China	Unknown	KT822253	—	KT822344	Guo et al. 2016
A. mellea-EA		*A. mellea*	89 07	strain	Japan	Unknown	AB510852	AB510808	AB510796	Hasegawa et al.
A. mellea-EA		*A. mellea*	94_10_1	strain	Japan	Unknown	AB510857	AB510813	AB510798	Hasegawa et al.
A. mellea-EA		*A. mellea*	94 68	strain	Japan	Unknown	AB510858	AB510814	AB510803	Hasegawa et al.
A. mellea-EA		*A. mellea*	94 7	strain	Japan	Unknown	AB510863	AB510819	AB510799	Hasegawa et al.
A. mellea-EA		*A. mellea*	97_6	strain	Japan	Unknown	AB510856	AB510812	AB510797	Hasegawa et al.
A. mellea-EA		*A. mellea*	A_10	strain	Japan	Unknown	AB510860	AB510816	AB510800	Hasegawa et al.
A. mellea-EA		*A. mellea*	A 12	strain	Japan	Unknown	AB510864	AB510820	AB510801	Hasegawa et al.
A. mellea-EA		*A. mellea*	94 5	strain	Japan	Unknown	AB510880	AB510833	AB510802	Ota et al. 2000
A. mellea-EA		CBS K[1]	CFCC 81073	single spore	Yunnan, China	Conifer and broadleaf forest	MG931746	MG931520	OL742429	this study
A. mellea-EA		/	HMAS253414	sporocarp	Yunnan, China	Broad leaved forest	MG931782	MG931556	OL742428	this study
A. mellea-EA		/	HMAS253585	sporocarp	Tibet, China	Conifer and broadleaf forest	MG931786	MG931560	OL742430	this study
A. mellea-EA		/	HMAS253587	sporocarp	Tibet, China	Conifer and broadleaf forest	MG931787	MG931561	OL742431	this study

A. mellea-EA	/	HMAS253605	sporocarp	Tibet, China	Conifer and broadleaf forest	MG931790	MG931564	OL742432	this study
A. mellea-EA	/	HMAS272369	sporocarp	Tibet, China	Rich Fir and Oak	MG931804	MG931578	MH002784	this study
A. mellea-EA	/	HMAS272919	sporocarp	Tibet, China	Conifer and broadleaf forest	MG931812	MG931586	OL742433	this study
A. mellea-EU	/	H7000666	sporocarp	Denmark	Unknown	MG931762	MG931536	MH002746	this study
A. mellea-EU	/	H7031840	sporocarp	France	Unknown	MG931767	MG931541	MH002751	this study
A. mellea-EU	*A. mellea*	D1	strain	France	Unknown	JN657464	JN657437	JN657491	Tsykun et al. 2013
A. mellea-EU	*A. mellea*	D4	strain	France	Unknown	HQ232290	HQ232282	HQ285904	Tsykun et al. 2013
A. mellea-EU	*A. mellea*	D5	strain	France	Unknown	JN657465	JN657438	JN657492	Tsykun et al. 2013
A. mellea-EU	*A. mellea*	HY3	strain	Ukraine	Unknown	JN657466	JN657439	JN657493	Tsykun et al. 2013
A. mellea-NA	*A. mellea*	ST20	sporocarp	Wisconsin, USA	Unknown	AY213586	AY509187	JF313128	Kim et al. 2006
A. mellea-NA	*A. mellea*	ST21	sporocarp	New Hampshire, USA	Unknown	AY213587	AY509188	JF313127	Kim et al. 2006
A. mellea-NA	*A. mellea*	ST5	sporocarp	Virginia, USA	Unknown	AY213585	AY509185	JF313137	Kim et al. 2006
A. mellea-NA	/	MIN0877204	sporocarp	Minnesota,USA	Unknown	MG931908	MG931682	—	this study
A. mellea-NA	/	TENN054336	sporocarp	Georgia, USA	Unknown	MG931920	MG931694	—	this study
A. mellea-NA	/	TENN061702	sporocarp	South Carolina, USA	Unknown	MG931929	MG931703	—	this study
A. sp.	/	HKAS 83303	rhizomorphs	Yunnan, China	*Gastradia elata*	KU378047	—	KT822437	Guo et al. 2016
A. sp.	/	HKAS 83361	rhizomorphs	Yunnan, China	*Gastradia elata*	KU378048	—	KT822436	Guo et al. 2016
A. sp.	/	HKAS 86541	rhizomorphs	Yunnan, China	*Gastradia elata*	KT822304	—	KT822435	Guo et al. 2016
A. borealis	*A. borealis*	HKAS 86616	tester strain	Helsinki, Finland	Unknown	KT822291	KT822427	KT822214	Guo et al. 2016
/	/	H6059109	sporocarp	Finland	On park lawn	MG931758	MG931532	MH002742	this study
/	/	H6059228	sporocarp	Finland	*Alnus incana*	MG931760	MG931534	MH002744	this study
/	/	HMJAU 22345	sporocarp	Belarus	Unknown	MG931834	MG931608	MH002821	this study
A. borealis	*A. borealis*	A1	strain	Finland	Unknown	JN657467	JN657440	JN657494	Tsykun et al. 2013
A. borealis	*A. borealis*	A2	strain	Finland	Unknown	HQ232287	HQ232279	HQ285901	Tsykun et al. 2013
A. borealis	*A. borealis*	A5	strain	Finland	Unknown	JN657468	JN657441	JN657495	Tsykun et al. 2013
A. borealis	*A. borealis*	A618	strain	Finland	Unknown	JN657469	JN657442	JN657496	Tsykun et al. 2013
A. sp.	CBS M[3]	CFCC 80932	single spore	Hubei, China	Conifer and broadleaf forest	MG931748	MG931522	MH002732	this study
A. sp.	CBS M[2]	CFCC 88644	single spore	Shaanxi, China	*Betula* sp.	MG931750	MG931524	MH002734	this study
A. sp.	CBS M[2]	CFCC 88884	single spore	Shaanxi, China	*Betula* sp.	MG931751	MG931525	MH002735	this study
A. sp.	CBS M[2]	CFCC 88886	single spore	Shaanxi, China	*Betula* sp.	MG931752	MG931526	MH002736	this study
A. gemina	*A. gemina*	ST8	sporocarp	New York, USA	Unknown	AY213555	AY509158	JF313136	Kim et al. 2006
A. gemina	*A. gemina*	ST9	sporocarp	New York, USA	Unknown	AY213556	AY509160	JF313135	Kim et al. 2006
A. sp.	/	TENN060293	sporocarp	North Carolina, USA	Unknown	MG931924	MG931698	MH002901	this study

A. sp.	/	TENN064035	sporocarp	Tennessee, USA	Unknown	MG931930	MG931704	MH002906	this study
A. sp.	/	TENN065407	sporocarp	Tennessee, USA	Unknown	MG931932	MG931706	MH002908	this study
A. sp.	/	H7031848	sporocarp	France	Rich *Abies alba* forest	MG931769	MG931543	MH002753	this study
A. ostoyae	*A. ostoyae*	BRNM 706815	strain	Czech Republic	*Fagus sylvatica*	EU257717	EU257711	EU251400	Antonín et al. 2009
A. sp.	CBS D[2]	CFCC 5024	single spore	Heilongjiang, China	Unknown	MG931718	MG931492	MH002705	this study
A. sp.	CBS D[2]	CFCC 5196	single spore	Jinlin, China	Unknown	MG931720	MG931494	MH002707	this study
A. sp.	CBS D[1]	CFCC 83492	single spore	Jinlin, China	*Pinus koraiensis*	MG931721	MG931495	MH002708	this study
A. sp.	/	HMJAU 25004	sporocarp	Jilin, China	Unknown	MG931835	MG931609	MH002816	this study
A. ostoyae	*A. ostoyae*	C2	strain	France	Unknown	JN657459	JN657432	JN657486	Tsykun et al. 2013
A. ostoyae	*A. ostoyae*	HpAg1	strain	Ukraine	Unknown	JN657462	JN657435	JN657489	Tsykun et al. 2013
A. ostoyae	*A. ostoyae*	C5	strain	France	Unknown	HQ232289	HQ232281	HQ285903	unknown
A. ostoyae	*A. ostoyae*	2002_66_03	strain	Japan	Unknown	AB510896	AB510847	AB510781	Hasegawa et al.
A. ostoyae	*A. ostoyae*	NC8	strain	Japan	Unknown	AB510897	AB510848	AB510782	Ota et al. 1998
A. sp.	CBS D[2]	CFCC 5195	single spore	Jinlin, China	Unknown	MG931719	MG931493	MH002706	this study
A. sp.	CBS D[2]	CFCC 83494	single spore	Inner Mongolia, China	Conifer and broadleaf forest	MG931722	MG931496	MH002709	this study
A. sp.	/	H7022044	sporocarp	Italy	Coniferous forest	MG931765	MG931539	MH002749	this study
A. sp.	/	H7031859	sporocarp	Germany	Unknown	MG931770	MG931544	MH002754	this study
A. sp.	/	HMJAU 29070	sporocarp	Jilin, China	Broad leaved forest	MG931836	MG931610	MH002817	this study
A. sp.	/	MIN0824275	sporocarp	Minnesota, USA	On oak roots.	MG931903	MG931677	MH002882	this study
A. sp.	/	TENN060929	sporocarp	Tennessee, USA	Unknown	MG931925	MG931699	MH002902	this study
A. sp.	/	TENN061648	sporocarp	Tennessee, USA	Unknown	MG931928	MG931702	MH002905	this study
A. sp.	/	TENN065195	sporocarp	North Carolina, USA	Unknown	MG931931	MG931705	MH002907	this study
A. sp.	/	HMAS145183	sporocarp	Shaanxi, China	Unknown	MG931778	MG931552	MH002762	this study
A. sp.	/	TENN055309	sporocarp	Krasnodar Krai Russia	Unknown	MG931921	MG931695	MH002898	this study
A. solidipes	*A. solidipes*	P1404	sporocarp	Idoale, USA	Unknown	AY213554	AY509157	JF313140	Kim et al. 2006
A. solidipes	*A. solidipes*	ST1	sporocarp	New Hemisphre USA	Unknown	AY213552	AY509154	JF313141	Kim et al. 2006
A. solidipes	*A. solidipes*	ST2	sporocarp	USA	Unknown	AY213553	AY509155	JF313139	Kim et al. 2006
A. nabsnona	*A. nabsnona*	C21	sporocarp	Idaho, USA	Unknown	AY213572	AY509175	JF313119	Kim et al. 2006
A. nabsnona	*A. nabsnona*	M90	sporocarp	British Columbia,	Unknown	AY213573	AY509177	JF313122	Kim et al. 2006
A. nabsnona	*A. nabsnona*	2000/3/1	strain	Japan	Unknown	AB510899	AB510850	AB510766	Ota et al. 2009
A. nabsnona	/	TENN052584	sporocarp	Washington USA	Unknown	MG931916	MG931690	MH002894	this study
PS I	*Nag. E*	94_35_01	strain	Japan	Unknown	AB510891	AB510843	AB510770	Ota et al. 1998
PS I	*Nag. E*	NE4	strain	Japan	Unknown	AB510874	AB510828	AB510771	Ota et al. 1998

PS I		/	HMAS253478	sporocarp	Tibet, China	*Rhododendron* sp.	MG931783	MG931557	MH002766	this study
PS II	CSP	/	HKAS 51046	sporocarp	Sichuan, China	Unknown	KT822257	—	KT822356	Guo et al. 2016
PS II	CSP	/	HKAS 85449	sporocarp	Yunnan, China	Unknown	KT822299	—	KT822387	Guo et al. 2016
PS II	CSP	/	HKAS 85457	sporocarp	Yunnan, China	Unknown	KT822271	—	KT822385	Guo et al. 2016
PS II	CSP	/	HKAS 85519	sporocarp	Yunnan, China	Unknown	KT822335	—	KT822406	Guo et al. 2016
PS II	CSP	/	HKAS 85527	sporocarp	Yunnan, China	Unknown	KT822322	—	KT822408	Guo et al. 2016
PS II	CSP	/	HKAS 85551	sporocarp	Yunnan, China	Unknown	KT822313	—	KT822382	Guo et al. 2016
PS II	CSP	/	HKAS 85575	sporocarp	Yunnan, China	Unknown	KT822314	—	KT822383	Guo et al. 2016
PS II	CSP	/	HKAS 85581	sporocarp	Yunnan, China	Unknown	KT822336	—	KT822392	Guo et al. 2016
PS II	CSP	/	HKAS 85594	sporocarp	Yunnan, China	Unknown	KT822274	—	KT822377	Guo et al. 2016
PS II	CSP	/	HKAS 86543	commercial strain	Yunnan, China	*Gastradia elata*	KT822334	—	KT822407	Guo et al. 2016
PS II	CSP	/	HKAS 86548	rhizomorphs	Yunnan, China	*Gastradia elata*	KT822296	—	KT822395	Guo et al. 2016
PS II	CSP	/	HKAS 86551	sporocarp	Yunnan, China	Unknown	KT822279	—	KT822368	Guo et al. 2016
PS II	CSP	/	HKAS 86552	rhizomorphs	Yunnan, China	*Gastradia elata*	KT822297	—	KT822393	Guo et al. 2016
PS II	CSP	/	HKAS 86553	sporocarp	Yunnan, China	Unknown	KT822298	—	KT822394	Guo et al. 2016
PS II	CSP	/	HKAS 86554	rhizomorphs	Yunnan, China	*Gastradia elata*	KT822260	—	KT822369	Guo et al. 2016
PS II	CSP	/	HKAS 86555	rhizomorphs	Yunnan, China	*Gastradia elata*	KT822302	—	KT822381	Guo et al. 2016
PS II	CSP	/	HKAS 86556	commercial strain	Yunnan, China	*Gastradia elata*	KT822259	—	KT822373	Guo et al. 2016
PS II	CSP	/	HKAS 86557	commercial strain	Yunnan, China	*Gastradia elata*	KT822301	—	KT822374	Guo et al. 2016
PS II	CSP	CBS C[1]	HKAS 86577	single spore	Heilongjiang, China	Unknown	KT822267	—	KT822379	Guo et al. 2016
PS II	CSP	CBS L[1]	HKAS 86613	single spore	Guizhou, China	Unknown	KT822319	—	KT822388	Guo et al. 2016
PS II	CSP	CBS L[1]	HKAS 86614	single spore	Guizhou, China	Unknown	KT822305	—	KT822391	Guo et al. 2016
PS II	CSP	CBS L[1]	HKAS 86615	single spore	Guizhou, China	Unknown	KT822315	—	KT822384	Guo et al. 2016
PS II	CSP	CBS O[1]	HKAS 86623	single spore	Sichuan, China	Unknown	KT822318	KM205355	KT822363	Guo et al. 2016
PS II	CSP	CBS C[1]	CFCC 83490	single spore	Jinlin, China	Conifer and broadleaf forest	MG931717	MG931491	MH002704	this study
PS II	CSP	CBS H[1]	CFCC 84786	single spore	Yunnan, China	Conifer and broadleaf forest	MG931731	MG931505	MH002718	this study
PS II	CSP	CBS J[3]	CFCC 84826	single spore	Xinjiang, China	Conifer and broadleaf forest	MG931734	MG931508	MH002719	this study
PS II	CSP	CBS J[4]	CFCC 84879	single spore	Shaanxi, China	Conifer and broadleaf forest	MG931735	MG931509	MH002720	this study
PS II	CSP	CBS J[2]	CFCC 84915	single spore	Shaanxi, China	Conifer and broadleaf forest	MG931736	MG931510	MH002721	this study
PS II	CSP	CBS J[2]	CFCC 88888	single spore	Hubei, China	*Abies* sp.	MG931737	MG931511	MH002722	this study
PS II	CSP	CBS J[4]	CFCC 88889	single spore	Shaanxi, China	*Betula* sp.	MG931738	MG931512	MH002723	this study
PS II	CSP	CBS J[4]	CFCC 88890	single spore	Shaanxi, China	*Betula* sp.	MG931739	MG931513	MH002724	this study
PS II	CSP	CBS J[4]	CFCC 88905	single spore	Shaanxi, China	*Acer* sp.	MG931740	MG931514	MH002725	this study

PS II	CSP	CBS J[4]	CFCC 88906	single spore	Shaanxi, China	*Acer* sp.	MG931741	MG931515	MH002726	this study
PS II	CSP	CBS J[4]	CFCC 88909	single spore	Shaanxi, China	*Quercus* sp.	MG931742	MG931516	MH002727	this study
PS II	CSP	CBS J[4]	CFCC 88910	single spore	Shaanxi, China	*Quercus* sp.	MG931743	MG931517	MH002728	this study
PS II	CSP	CBS N[1]	CFCC 88927	single spore	Sichuan, China	*Castanopsis sempervirens*	MG931753	MG931527	MH002737	this study
PS II	CSP	CBS N[1]	CFCC 88928	single spore	Sichuan, China	*Castanopsis sempervirens*	MG931754	MG931528	MH002738	this study
PS II	CSP	CBS N[4]	CFCC 88938	single spore	Shaanxi, China	*Acer* sp.	MG931755	MG931529	MH002739	this study
PS II	CSP	CBS N[4]	CFCC 88939	single spore	Shaanxi, China	*Acer* sp.	MG931756	MG931530	MH002740	this study
PS II	CSP	/	HMAS130389	sporocarp	Qinghai, China	Unknown	MG931772	MG931546	MH002756	this study
PS II	CSP	/	HMAS130445	sporocarp	Qinghai, China	Unknown	MG931773	MG931547	MH002757	this study
PS II	CSP	/	HMAS130491	sporocarp	Qinghai, China	Unknown	MG931774	MG931548	MH002758	this study
PS II	CSP	/	HMAS130603	sporocarp	Qinghai, China	Unknown	MG931775	MG931549	MH002759	this study
PS II	CSP	/	HMAS132012	sporocarp	Qinghai, China	Coniferous forest	MG931776	MG931550	MH002760	this study
PS II	CSP	/	HMAS132015	sporocarp	Qinghai, China	Poplar	MG931777	MG931551	MH002761	this study
PS II	CSP	/	HMAS154598	sporocarp	Qinghai, China	Unknown	MG931779	MG931553	MH002763	this study
PS II	CSP	/	HMAS253383	sporocarp	Xinjiang, China	*Picea abies*	MG931781	MG931555	MH002765	this study
PS II	CSP	/	HMAS253500	sporocarp	Tibet, China	Unknown	MG931784	MG931558	MH002767	this study
PS II	CSP	/	HMAS253588	sporocarp	Tibet, China	Fir	MG931788	MG931562	MH002769	this study
PS II	CSP	/	HMAS253595	sporocarp	Tibet, China	Fir	MG931789	MG931563	MH002770	this study
PS II	CSP	/	HMAS253629	sporocarp	Tibet, China	Conifer and broadleaf forest	MG931791	MG931565	MH002771	this study
PS II	CSP	/	HMAS253848	sporocarp	Beijing, China	Unknown	MG931796	MG931570	MH002776	this study
PS II	CSP	/	HMAS264994	sporocarp	Tibet, China	*Rhododendron* sp.	MG931798	MG931572	MH002778	this study
PS II	CSP	/	HMAS267155	sporocarp	Beijing, China	Unknown	MG931799	MG931573	MH002779	this study
PS II	CSP	/	HMAS267218	sporocarp	Beijing, China	Unknown	MG931800	MG931574	MH002780	this study
PS II	CSP	/	HMAS268710	sporocarp	Sichuan, China	*Pinus* sp.	MG931801	MG931575	MH002781	this study
PS II	CSP	/	HMAS270241	sporocarp	Xinjiang, China	*Spruce* sp.	MG931802	MG931576	MH002782	this study
PS II	CSP	/	HMAS272390	sporocarp	Tibet, China	Rich Fir and Oak	MG931805	MG931579	MH002785	this study
PS II	CSP	/	HMAS272485	sporocarp	Tibet, China	Fir	MG931806	MG931580	MH002786	this study
PS II	CSP	/	HMAS272491	sporocarp	Tibet, China	Unknown	MG931807	MG931581	MH002787	this study
PS II	CSP	/	HMAS272515	sporocarp	Tibet, China	Broad leaved forest	MG931808	MG931582	MH002788	this study
PS II	CSP	/	HMAS272656	sporocarp	Tibet, China	Conifer and broadleaf forest	MG931809	MG931583	MH002789	this study
PS II	CSP	/	HMAS272891	sporocarp	Tibet, China	Fir	MG931810	MG931584	MH002790	this study
PS II	CSP	/	HMAS272905	sporocarp	Tibet, China	Fir	MG931811	MG931585	MH002791	this study
PS II	CSP	/	HMAS273634	sporocarp	Beijing, China	Unknown	MG931815	MG931589	MH002794	this study

PS II	CSP	/	HMAS274072	sporocarp	Tibet, China	Unknown	MG931817	MG931591	MH002796	this study
PS II	CSP	/	HMAS274337	sporocarp	Tibet, China	Rich Pinus and Rosaceous	MG931818	MG931592	MH002797	this study
PS II	CSP	/	HMAS274342	sporocarp	Tibet, China	Rich Pinus and Rosaceous	MG931819	MG931593	MH002798	this study
PS II	CSP	/	HMAS274349	sporocarp	Sichuan, China	Fir	MG931820	MG931594	MH002799	this study
PS II	CSP	/	HMAS274375	sporocarp	Sichuan, China	Fir	MG931821	MG931595	MH002800	this study
PS II	CSP	/	HMAS274442	sporocarp	Sichuan, China	Rich Fir and Oak forest	MG931822	MG931596	MH002801	this study
PS II	CSP	/	HMAS274553	sporocarp	Sichuan, China	*Betula* sp.	MG931823	MG931597	MH002802	this study
PS II	CSP	/	HMAS280003	sporocarp	Tibet, China	Fir	MG931824	MG931598	MH002803	this study
PS II	CSP	/	HMAS280006	sporocarp	Tibet, China	Fir	MG931825	MG931599	MH002804	this study
PS II	CSP	/	HMAS280010	sporocarp	Tibet, China	Unknown	MG931826	MG931600	MH002805	this study
PS II	CSP	/	HMAS5075	sporocarp	Tibet, China	Unknown	MG931827	MG931601	MH002806	this study
PS II	CSP	/	HMAS96843	sporocarp	Qinghai, China	*Betula* sp.	MG931829	MG931603	MH002808	this study
PS II	CSP	/	HMAS99054	sporocarp	Xinjiang, China	Unknown	MG931831	MG931605	MH002810	this study
PS II	CSP	/	HMAS99353	sporocarp	Qinghai, China	Unknown	MG931832	MG931606	MH002811	this study
PS II	CSP	/	HMAS99623	sporocarp	Qinghai, China	Unknown	MG931833	MG931607	MH002812	this study
PS II	CSP	/	HMJAU24389	sporocarp	Heilongjiang, China	Spruce	MG931839	MG931613	MH002815	this study
PS II	CSP	/	HMJAU35880	sporocarp	Qinghai, China	Unknown	MG931840	MG931614	MH002818	this study
PS II	CSP	/	HMJAU5534	sporocarp	Xinjiang, China	Unknown	MG931841	MG931615	MH002819	this study
PS II	CSP	/	HMJAU5541	sporocarp	Xinjiang, China	Broad leaved forest	MG931842	MG931616	MH002820	this study
PS II	Sin-Cep	CBS F[1]	CFCC 84778	single spore	Liaoning, China	*Betula* sp.	MG931724	MG931498	MH002711	this study
PS II	Sin-Cep	CBS F[1]	CFCC 85239	single spore	Liaoning, China	Conifer and broadleaf forest	MG931727	MG931501	MH002714	this study
PS II	Sin-Cep	CBS F[1]	CFCC 88920	single spore	Jinlin, China	*Acer* sp.	MG931728	MG931502	MH002715	this study
PS II	Sin-Cep	CBS F[2]	CFCC 88921	single spore	Hubei, China	*Betula* sp.	MG931729	MG931503	MH002716	this study
PS II	Sin-Cep	CBS F[2]	CFCC 88924	single spore	Shaanxi, China	*Tilia* sp.	MG931730	MG931504	MH002717	this study
PS II	Sin-Cep	*A. cepistipes*	BRNM 706814	sporocarp	Czech Republic	Fagus sylvatica	EU257715	EU257709	EU251395	Antonín et al. 2009
PS II	Sin-Cep	*A. cepistipes*	BRNM 695717	sporocarp	Slovakia	*Picea abies*	EU257716	EU257710	EU251396	Antonín et al. 2009
PS II	Sin-Cep	*A. cepistipes*	CMW31082	strain	Finland	Unknown	—	KM205310	KM205257	Coetzee et al. 2015
PS II	Sin-Cep	*A. cepistipes*	CMW31083	strain	Italy	Unknown	—	KM205311	KM205258	Coetzee et al. 2015
PS II	Sin-Cep	CBS F[1]	HKAS 86583	single spore	Heilongjiang, China	Unknown	KT822290	—	KT822417	Guo et al. 2016
PS II	Sin-Cep	CBS F[1]	HKAS 86584	single spore	Liaoning, China	Unknown	KT822278	—	KT822419	Guo et al. 2016
PS II	Sin-Cep	*A. cepistipes*	2000_07_04	strain	Japan	Unknown	AB510870	AB510825	AB510788	Hasegawa et al.
PS II	Sin-Cep	*A. cepistipes*	1999-528	strain	Finland	Unknown	—	KJ414316	KJ414319	Keča et al. 2015
PS II	Sin-Cep	*A. cepistipes*	1999-531	strain	Italy	Unknown	—	KJ414318	KJ414321	Keča et al. 2015

PS II	Sin-Cep	*A. cepistipes*	LLC	strain	UK	Unknown	—	JF288719	JF746913	Keča et al. 2015
PS II	Sin-Cep	*A. cepistipes*	RHB1	strain	UK	Unknown	—	JF288744	JF746914	Keča et al. 2015
PS II	Sin-Cep	*A. cepistipes*	RHB2	strain	UK	Unknown	—	JF288745	JF746915	Keča et al. 2015
PS II	Sin-Cep	*A. cepistipes*	RHB3	strain	UK	Unknown	—	JF288746	JF746916	Keča et al. 2015
PS II	Sin-Cep	*A. cepistipes*	SY1RA	strain	UK	Unknown	—	JF288720	JF746917	Keča et al. 2015
PS II	Sin-Cep	*A. cepistipes*	ND1	strain	Japan	Unknown	AB510885	AB510837	AB510792	Ota et al. 1998
PS II	Sin-Cep	CBS F[1]	CFCC 84781	single spore	Heilongjiang, China	Unknown	MG931725	MG931499	MH002712	this study
PS II	Sin-Cep	/	CFCC 85691	single spore	Liaoning, China	Broad leaved forest	MG931747	MG931521	MH002731	this study
PS II	Sin-Cep	CBS F[2]	CFCC 88925	single spore	Shaanxi, China	*Tilia* sp.	MG931726	MG931500	MH002713	this study
PS II	Sin-Cep	/	H6059220	sporocarp	Finland	Unknown	MG931759	MG931533	MH002743	this study
PS II	Sin-Cep	/	H6059240	sporocarp	Finland	Rich *Abies alba* forest	MG931761	MG931535	MH002745	this study
PS II	Sin-Cep	/	H7019812	sporocarp	France	*Picea abies*	MG931764	MG931538	MH002748	this study
PS II	Sin-Cep	/	H7028498	sporocarp	Russia	*Tilia* sp.	MG931766	MG931540	MH002750	this study
PS II	Sin-Cep	/	H7031847	sporocarp	Norway	Unknown	MG931768	MG931542	MH002752	this study
PS II	Sin-Cep	/	HMAS253828	sporocarp	Beijing, China	Unknown	MG931795	MG931569	MH002775	this study
PS II	Sin-Cep	/	HMAS270859	sporocarp	Beijing, China	Rich Pinus and Rosaceous plant	MG931803	MG931577	MH002783	this study
PS II	Sin-Cep	/	HMAS273636	sporocarp	Beijing, China	Unknown	MG931816	MG931590	MH002795	this study
PS II	Sin-Cep	/	HMAS72996	sporocarp	Finland, China	Populus	MG931828	MG931602	MH002807	this study
PS II	Sin-Cep	/	HMJAU23629	sporocarp	Inner Mongolia, China	Unknown	MG931837	MG931611	MH002813	this study
PS II	Sin-Cep	/	HMJAU23939	sporocarp	Jilin, China	Unknown	MG931838	MG931612	MH002814	this study
PS II	Sin-Cep	/	TENN049335	sporocarp	Primorsky Krai, Russia	Unknown	MG931913	MG931687	MH002891	this study
PS II	Sin-Cep	/	TENN049477	sporocarp	Primorsky Krai, Russia	Unknown	MG931914	MG931688	MH002892	this study
PS II	Sin-Cep	*A. cepistipes*	C5CS1	strain	Ukarine	Unknown	JN657450	JN657423	JN657477	Tsykun et al. 2013
PS II	Sin-Cep	*A. cepistipes*	S11AE	strain	Ukarine	Unknown	JN657451	JN657424	JN657478	Tsykun et al. 2013
PS II	Sin-Cep	*A. cepistipes*	Y16AE	strain	Ukarine	Unknown	JN657449	JN657422	JN657476	Tsykun et al. 2013
PS II	Sin-Cep	CBS A[1]	HKAS 86566	single spore	Jilin, China	Unknown	KT822323	—	KT822422	Guo et al. 2016
PS II	Sin-Cep	CBS A[1]	HKAS 86567	single spore	Jilin, China	Unknown	KT822272	—	KT822423	Guo et al. 2016
PS II	Sin-Cep	CBS A[1]	HKAS 86568	single spore	Heilongjiang, China	Unknown	KT822262	—	KT822424	Guo et al. 2016
PS II	Sin-Cep	*A. sinapina*	M50	sporocarp	British Columbia,	Unknown	AY213564	AY509167	JF313114	Kim et al. 2006
PS II	Sin-Cep	*A. sinapina*	ST12	sporocarp	Washington, USA	Unknown	AY213565	AY509168	JF313132	Kim et al. 2006
PS II	Sin-Cep	*A. sinapina*	ST13	sporocarp	Michigan, USA	Unknown	AY213566	AY509169	JF313131	Kim et al. 2006
PS II	Sin-Cep	*A. sinapina*	05_13_2	strain	Japan	Unknown	AB510884	AB510836	AB510776	Ota et al. 2009
PS II	Sin-Cep	*A. sinapina*	96_7_1	strain	Japan	Unknown	AB510873	AB510827	AB510774	Ota et al. 2009

PS II	Sin-Cep	CBS A[1]	CFCC 80965	single spore	Jinlin, China	*Betula ermanii*, conifer and	MG931712	MG931486	MH002699	this study
PS II	Sin-Cep	CBS A[1]	CFCC 83495	single spore	Jinlin, China	*Betula ermanii*, conifer and	MG931713	MG931487	MH002700	this study
PS II	Sin-Cep	/	HMAS96969	sporocarp	Jilin, China	Unknown	MG931830	MG931604	MH002809	this study
PS II	Sin-Cep	/	MIN0896233	sporocarp	Minnesota, USA	On well rotted mossy log on	MG931909	MG931683	MH002887	this study
PS II	Sin-Cep	/	MIN925920	sporocarp	Minnesota, USA	Unknown	MG931912	MG931686	MH002890	this study
PS II	Sin-Cep	/	TENN049521	sporocarp	Primorsky Krai, Russia	Unknown	MG931915	MG931689	MH002893	this study
PS II	Sin-Cep	/	TENN052598	sporocarp	British Columbia,	Unknown	MG931917	MG931691	MH002895	this study
PS II	Sin-Cep	/	TENN054249	sporocarp	Alaska, USA	Unknown	MG931918	MG931692	MH002896	this study
PS II	Sin-Cep	/	TENN054251	sporocarp	Alaska, USA	Unknown	MG931919	MG931693	MH002897	this study
PS II	Sin-Cep	/	TENN056893	sporocarp	Alaska USA	Unknown	MG931922	MG931696	MH002899	this study
PS II	Sin-Cep	/	WTU550665	sporocarp	Alaska, USA	Unknown	MG931935	MG931709	MH002911	this study
PS II	Sin-Cep	/	WTU563903	sporocarp	Alaska, USA	Unknown	MG931937	MG931711	MH002913	this study
PS II	Gallica	/	HKAS 45821	sporocarp	Yunnan, China	Unknown	KT822261	—	KT822396	Guo et al. 2016
PS II	Gallica	/	HKAS 85567	sporocarp	Yunnan, China	Unknown	KT822268	—	KT822396	Guo et al. 2016
PS II	Gallica	/	HKAS 86558	commercial strain	Korea	*Gastradia elata*	KT822285	—	KT822405	Guo et al. 2016
PS II	Gallica	/	HKAS 86559	commercial strain	Shaanxi, China	*Gastradia elata*	KT822289	—	KT822399	Guo et al. 2016
PS II	Gallica	/	HKAS 86560	commercial strain	Shaanxi, China	*Gastradia elata*	KT822264	—	KT822401	Guo et al. 2016
PS II	Gallica	/	HKAS 86563	sporocarp	Hubei, China	Unknown	KT822286	—	KT822397	Guo et al. 2016
PS II	Gallica	/	HKAS 86564	commercial strain	Hubei, China	*Gastradia elata*	KT822284	—	KT822397	Guo et al. 2016
PS II	Gallica	*A. gallica*	84 088	strain	Germany	Unknown	—	KJ200945	KJ200953	Keča et al. 2015
PS II	Gallica	*A. gallica*	86 016	strain	Germany	Unknown	—	KJ200946	KJ200952	Keča et al. 2015
PS II	Gallica	*A. gallica*	86 032	strain	Germany	Unknown	—	KJ200949	KJ200955	Keča et al. 2015
PS II	Gallica	*A. gallica*	NA13	strain	Japan	Unknown	AB510890	AB510842	AB510760	Ota et al. 1998
PS II	Gallica	*A. gallica*	NA4	strain	Japan	Unknown	AB510881	AB510834	AB510761	Ota et al. 1998
PS II	Gallica	/	CFCC 80379	sporocarp	Sichuan, China	Unknown	MG931744	MG931518	MH002729	this study
PS II	Gallica	/	CFCC 80383	rhizomorphs	Shaanxi, China	Unknown	MG931745	MG931519	MH002730	this study
PS II	Gallica	CBS B[2]	CFCC 80936	single spore	Heilongjiang, China	Conifer and broadleaf forest	MG931714	MG931488	MH002701	this study
PS II	Gallica	CBS B[1]	CFCC 80976	single spore	Jinlin, China	Conifer and broadleaf forest	MG931715	MG931489	MH002702	this study
PS II	Gallica	CBS B[2]	CFCC 81053	single spore	Liaoning, China	Conifer and broadleaf forest	MG931716	MG931490	MH002703	this study
PS II	Gallica	/	CFCC 81434	sporocarp	Liaoning, China	Unknown	MG931749	MG931523	MH002733	this study
PS II	Gallica	/	CFCC 88346	sporocarp	Jinlin, China	Oak	MG931723	MG931497	MH002710	this study
PS II	Gallica	/	HMAS253529	sporocarp	Tibet, China	Fir	MG931785	MG931559	MH002768	This study
PS II	Gallica	/	HMAS253805	sporocarp	Beijing, China	Unknown	MG931792	MG931566	MH002772	this study

PS II	Gallica	/	HMAS253807	sporocarp	Beijing, China	Unknown	MG931793	MG931567	MH002773	this study
PS II	Gallica	/	HMAS253808	sporocarp	Beijing, China	Unknown	MG931794	MG931568	MH002774	this study
PS II	Gallica	/	HMAS253866	sporocarp	Beijing, China	Unknown	MG931797	MG931571	MH002777	this study
PS II	Gallica	/	HMAS273624	sporocarp	Beijing, China	Unknown	MG931813	MG931587	MH002792	this study
PS II	Gallica	/	HMAS273625	sporocarp	Beijing, China	Unknown	MG931814	MG931588	MH002793	this study
PS II	Gallica	/	LC7345	commercial strain	Shaanxi, China	*Polyporus umbellatous*	MG931844	MG931618	MH002823	this study
PS II	Gallica	/	LC7543	single spore	Heilongjiang, China	Broad leaved forest	MG931845	MG931619	MH002824	this study
PS II	Gallica	/	LC7604	single spore	Heilongjiang, China	Broad leaved forest	MG931846	MG931620	MH002825	this study
PS II	Gallica	/	LC7626	single spore	Heilongjiang, China	Broad leaved forest	MG931847	MG931621	MH002826	this study
PS II	Gallica	/	LC7630	single spore	Heilongjiang, China	Broad leaved forest	MG931848	MG931622	MH002827	this study
PS II	Gallica	/	LC7631	single spore	Heilongjiang, China	Broad leaved forest	MG931849	MG931623	MH002828	this study
PS II	Gallica	/	LC7633	single spore	Heilongjiang, China	Broad leaved forest	MG931850	MG931624	MH002829	this study
PS II	Gallica	/	LC7634	single spore	Heilongjiang, China	Broad leaved forest	MG931851	MG931625	MH002830	this study
PS II	Gallica	/	LC7635	single spore	Heilongjiang, China	Broad leaved forest	MG931852	MG931626	MH002831	this study
PS II	Gallica	/	LC7636	single spore	Heilongjiang, China	Broad leaved forest	MG931853	MG931627	MH002832	this study
PS II	Gallica	/	LC7637	single spore	Heilongjiang, China	Broad leaved forest	MG931854	MG931628	MH002833	this study
PS II	Gallica	/	LC7638	single spore	Heilongjiang, China	Broad leaved forest	MG931855	MG931629	MH002834	this study
PS II	Gallica	/	LC7639	single spore	Heilongjiang, China	Broad leaved forest	MG931856	MG931630	MH002835	this study
PS II	Gallica	/	LC7641	single spore	Heilongjiang, China	Broad leaved forest	MG931857	MG931631	MH002836	this study
PS II	Gallica	/	LC7645	single spore	Heilongjiang, China	Broad leaved forest	MG931858	MG931632	MH002837	this study
PS II	Gallica	/	LC7650	single spore	Heilongjiang, China	Broad leaved forest	MG931859	MG931633	MH002838	this study
PS II	Gallica	/	LC7652	single spore	Heilongjiang, China	Broad leaved forest	MG931860	MG931634	MH002839	this study
PS II	Gallica	/	LC7653	single spore	Heilongjiang, China	Broad leaved forest	MG931861	MG931635	MH002840	this study
PS II	Gallica	/	LC7656	single spore	Heilongjiang, China	Broad leaved forest	MG931862	MG931636	MH002841	this study
PS II	Gallica	/	LC7666	single spore	Heilongjiang, China	Broad leaved forest	MG931863	MG931637	MH002842	this study
PS II	Gallica	/	LC7669	single spore	Heilongjiang, China	Broad leaved forest	MG931864	MG931638	MH002843	this study
PS II	Gallica	/	LC7671	single spore	Heilongjiang, China	Broad leaved forest	MG931865	MG931639	MH002844	this study
PS II	Gallica	/	LC7723	single spore	Heilongjiang, China	Broad leaved forest	MG931866	MG931640	MH002845	this study
PS II	Gallica	/	LC7725	single spore	Heilongjiang, China	Broad leaved forest	MG931867	MG931641	MH002846	this study
PS II	Gallica	/	LC7727	single spore	Heilongjiang, China	Broad leaved forest	MG931868	MG931642	MH002847	this study
PS II	Gallica	/	LC7729	single spore	Heilongjiang, China	Broad leaved forest	MG931869	MG931643	MH002848	this study
PS II	Gallica	/	LC7731	single spore	Shaanxi, China	*Polyporus umbellatous*	MG931843	MG931617	MH002822	this study
PS II	Gallica	/	LC7734	single spore	Heilongjiang, China	Broad leaved forest	MG931870	MG931644	MH002849	this study

PS II	Gallica	/	LC7737	single spore	Heilongjiang, China	Broad leaved forest	MG931871	MG931645	MH002850	this study
PS II	Gallica	/	LC7744	single spore	Heilongjiang, China	Broad leaved forest	MG931872	MG931646	MH002851	this study
PS II	Gallica	/	LC7745	single spore	Heilongjiang, China	Broad leaved forest	MG931873	MG931647	MH002852	this study
PS II	Gallica	/	LC7746	single spore	Heilongjiang, China	Broad leaved forest	MG931874	MG931648	MH002853	this study
PS II	Gallica	/	LC7747	single spore	Heilongjiang, China	Broad leaved forest	MG931875	MG931649	MH002854	this study
PS II	Gallica	/	LC7749	single spore	Heilongjiang, China	Broad leaved forest	MG931876	MG931650	MH002855	this study
PS II	Gallica	/	LC7765	single spore	Heilongjiang, China	Broad leaved forest	MG931877	MG931651	MH002856	this study
PS II	Gallica	/	LC7767	single spore	Heilongjiang, China	Broad leaved forest	MG931878	MG931652	MH002857	this study
PS II	Gallica	/	LC7770	single spore	Heilongjiang, China	Broad leaved forest	MG931879	MG931653	MH002858	this study
PS II	Gallica	/	LC7771	single spore	Heilongjiang, China	Broad leaved forest	MG931884	MG931658	MH002863	this study
PS II	Gallica	/	LC7772	single spore	Heilongjiang, China	Broad leaved forest	MG931880	MG931654	MH002859	this study
PS II	Gallica	/	LC7830	rhizomorphs	Yunnan, China	*Gastradia elata*	MG931881	MG931655	MH002860	this study
PS II	Gallica	/	LC7834	sporocarp	Yunnan, China	*Gastradia elata*	MG931882	MG931656	MH002861	this study
PS II	Gallica	/	LC7836	rhizomorphs	Yunnan, China	*Gastradia elata*	MG931883	MG931657	MH002862	this study
PS II	Gallica	/	LJM818	rhizomorphs	Yunnan, China	*Gastradia elata*	MG931885	MG931659	MH002864	this study
PS II	Gallica	/	LJM819	rhizomorphs	Yunnan, China	*Gastradia elata*	MG931886	MG931660	MH002865	this study
PS II	Gallica	/	LJM820	rhizomorphs	Yunnan, China	*Gastradia elata*	MG931887	MG931661	MH002866	this study
PS II	Gallica	/	LJM821	rhizomorphs	Yunnan, China	*Gastradia elata*	MG931888	MG931662	MH002867	this study
PS II	Gallica	/	LJM822	rhizomorphs	Yunnan, China	*Gastradia elata*	MG931889	MG931663	MH002868	this study
PS II	Gallica	/	LJM823	rhizomorphs	Yunnan, China	*Gastradia elata*	MG931890	MG931664	MH002869	this study
PS II	Gallica	/	LJM824	rhizomorphs	Yunnan, China	*Gastradia elata*	MG931891	MG931665	MH002870	this study
PS II	Gallica	/	LJM825	sporocarp	Heilongjiang, China	Broad leaved forest	MG931892	MG931666	MH002871	this study
PS II	Gallica	/	LJM826	rhizomorphs	Yunnan, China	*Gastradia elata*	MG931893	MG931667	MH002872	this study
PS II	Gallica	/	LJM827	rhizomorphs	Yunnan, China	*Gastradia elata*	MG931894	MG931668	MH002873	this study
PS II	Gallica	/	LJM828	sporocarp	Heilongjiang, China	Broad leaved forest	MG931895	MG931669	MH002874	this study
PS II	Gallica	/	LJM829	rhizomorphs	Yunnan, China	*Gastradia elata*	MG931896	MG931670	MH002875	this study
PS II	Gallica	/	LJM830	rhizomorphs	Yunnan, China	*Gastradia elata*	MG931897	MG931671	MH002876	this study
PS II	Gallica	/	LJM831	sporocarp	Heilongjiang, China	Broad leaved forest	MG931898	MG931672	MH002877	this study
PS II	Gallica	/	LJM832	commercial strain	Anhui, China	*Polyporus umbellatus*	MG931899	MG931673	MH002878	this study
PS II	Gallica	/	LJM833	sporocarp	Heilongjiang, China	Broad leaved forest	MG931900	MG931674	MH002879	this study
PS II	Gallica	/	LJM834	sporocarp	Heilongjiang, China	Broad leaved forest	MG931901	MG931675	MH002880	this study
PS II	Gallica	/	LJM835	single spore	Heilongjiang, China	Broad leaved forest	MG931902	MG931676	MH002881	this study
PS II	Gallica	*A. gallica*	ST22	sporocarp	Michigan, USA	Unknown	AY213569	AY509172	JF313126	Kim et al. 2006

PS II	Gallica	*A. gallica*	ST23	sporocarp	Wisconsin, USA	Unknown	AY213571	AY509173	JF313125	Kim et al. 2006
PS II	Gallica	*A. calvescens*	ST17	sporocarp	Michigan, USA	Unknown	AY213561	AY509164	JF895836	Kim et al. 2006
PS II	Gallica	*A. calvescens*	ST18	sporocarp	Michigan, USA	Unknown	AY213562	AY509165	JF313129	Kim et al. 2006
PS II	Gallica	*A. calvescens*	ST3	sporocarp	Quebec, Canada	Unknown	AY213559	AY509163	JF895835	Kim et al. 2006
PS II	Gallica	/	MIN0830453	sporocarp	Minnesota, USA	On ground, mainly under red	MG931904	MG931678	MH002883	this study
PS II	Gallica	/	MIN0830455	sporocarp	Minnesota, USA	On ground, mainly under red	MG931905	MG931679	MH002884	this study
PS II	Gallica	/	MIN0831333	sporocarp	Minnesota, USA	Under red oak, elm, and poplar.	MG931906	MG931680	MH002885	this study
PS II	Gallica	/	MIN0876971	sporocarp	Minnesota, USA	Oak savanna	MG931907	MG931681	MH002886	this study
PS II	Gallica	/	MIN0896482	sporocarp	Minnesota, USA	On ground under maple and	MG931910	MG931684	MH002888	this study
PS II	Gallica	/	MIN925752	sporocarp	Minnesota, USA	Unknown	MG931911	MG931685	MH002889	this study
PS II	Gallica	/	TENN060282	sporocarp	North Carolina USA	Unknown	MG931923	MG931697	MH002900	this study
PS II	Gallica	/	TENN061154	sporocarp	North Carolina, USA	Unknown	MG931926	MG931700	MH002903	this study
PS II	Gallica	/	TENN061319	sporocarp	North Carolina, USA	Unknown	MG931927	MG931701	MH002904	this study
PS II	Gallica	/	TENN065907	sporocarp	Mississippi,USA	Unknown	MG931933	MG931707	MH002909	this study
PS II	Gallica	/	TENN070870	sporocarp	North Carolina, USA	Unknown	MG931934	MG931708	MH002910	this study
PS II	Gallica	/	WTU533440	sporocarp	Idaho, USA	Unknown	MG931936	MG931710	MH002912	this study
PS II	Gallica	*A. altimontana*	837	strain	Idaho, USA	Unknown	AY213575	AY509179	JF313120	Kim et al. 2006
PS II	Gallica	*A. altimontana*	D82	sporocarp	Idaho, USA	Unknown	AY213577	JX459569	JF313118	Kim et al. 2006
PS II	Gallica	*A. altimontana*	POR100	sporocarp	Idaho, USA	Unknown	AY213579	AY509181	JF313117	Kim et al. 2006
PS II	Gallica	*A. cepistipes*	M110	sporocarp	British Columbia,	Unknown	AY213581	AY509182	JF313121	Kim et al. 2006
PS II	Gallica	*A. cepistipes*	S20	sporocarp	USA	Unknown	AY213582	AY509183	JF313116	Kim et al. 2006
PS II	Gallica	*A. cepistipes*	W113	sporocarp	Washington, USA	Unknown	AY213583	AY509184	JF313115	Kim et al. 2006
PS II	Gallica	*A. gallica*	EE4	strain	France	*Rubus fruticosus*	—	JF288736	JF746919	Mulholland et al.
PS II	Gallica	*A. gallica*	EE5	strain	France	*Corylus avellana*	—	JF288737	JF746920	Mulholland et al.
PS II	Gallica	*A. gallica*	EE6	strain	France	Unknown	—	JF288738	JF746921	Mulholland et al.
PS II	Gallica	*A. gallica*	HMAS251030	sporocarp	Italy, China	Unknown	MG931780	MG931554	MH002764	this study
PS II	Gallica	*A. gallica*	E4	strain	France	Unknown	JN657452	JN657425	JN657479	Tsykun et al. 2013
PS II	Gallica	*A. gallica*	E6	strain	France	Unknown	JN657453	JN657426	JN657480	Tsykun et al. 2013
PS II	Gallica	*A. gallica*	HY2a	strain	Ukraine	Unknown	JN657455	JN657428	JN657482	Tsykun et al. 2013
PS II	Gallica	*A. gallica*	Y7CS1	strain	Ukraine	Unknown	—	JN657431	JN657485	Tsykun et al. 2013
PS II	Gallica	/	H7005664	sporocarp	United States	*Acer* sp.	MG931763	MG931537	MH002747	this study
PS II	Gallica	/	H7031865	sporocarp	United States	Unknown	MG931771	MG931545	MH002755	this study
PS II	Gallica	*A. gallica*	HKAS 86569	tester strain	Europe	Unknown	KT822277	—	KT822414	Guo et al. 2016

[*] Three populations detected in PS II.

[1] assigned to biological species in Qin et al.(2007) based on intersterility tests.

[2] identified by Zhao Jun (General station of Forest Pest Control, State Forestry Administration) by intersterility tests.

[3] assigned to biological species in Zhao et al. (2007) based on intersterility tests.

[4] assigned to biological species in Wang (2007) based on intersterility tests.
