# Supplementary material for: Phylogenetic Relationships, Speciation, and Origin of Armillaria in the Northern Hemisphere: A Lesson Based on rRNA and Elongation Factor 1-Alpha
Source: J Fungi (Basel). 2021 Dec 17;7(12):1088. doi: 10.3390/jof7121088 (PMC8705980; doi:10.3390/jof7121088)
Supplement: Supplementary file 1 [file jof-07-01088-s001.zip › supplementary files/Table S2 Molecular Clock.pdf]

Table S2 Species and their sequences used for molecular clock analysis.

Class	Order	Clade	Family	Species	Isolate ID	ITS	TEF-1α
Agaricomycetes	Agaricales	Marasmioid	Omphalotaceae	*Gymnopus confluens*	BRNM734005	JX536124	JX536178
Agaricomycetes	Agaricales	Marasmioid	Omphalotaceae	*Anthracophyllum archeri*	DQ404387	DQ404387	DQ028586
Agaricomycetes	Agaricales	Marasmioid	Marasmiaceae	*Marasmius alliaceus*	AFTOL-ID 556	AY854076	AY883430
Agaricomycetes	Agaricales	Marasmioid	Hydropoid	*Mycena pura*	CBH216	FN394598	KF723616
Agaricomycetes	Agaricales	Marasmioid	Cyphellaceae	*Chondrostereum purpureum*	AFTOL-ID 441	DQ200929	DQ457632
Agaricomycetes	Agaricales	Marasmioid	Physalacriaceae	*Physalacria bambusae*	CBS712_83	DQ097367	GU187732
Agaricomycetes	Agaricales	Marasmioid	Physalacriaceae	*Xerula sinopudens*	HKAS_56793	KF530550	KF530579
Agaricomycetes	Agaricales	Marasmioid	Physalacriaceae	*Cyptotrama* sp.	RAK-2015	KU170956	KU289106
Agaricomycetes	Agaricales	Marasmioid	Physalacriaceae	*Flammulina velutipes*	AFTOL-ID 558	AY854073	AY883423
Agaricomycetes	Agaricales	Marasmioid	Physalacriaceae	*Cylindrobasidium laeve*	AFTOL-ID 453	DQ205682	DQ408151
Agaricomycetes	Agaricales	Marasmioid	Physalacriaceae	*Guyanagaster necrorhizus*	RAK31	KU170948	KU289108
Agaricomycetes	Agaricales	Marasmioid	Physalacriaceae	*Desarmillaria ectypa*	BRNM704974	EU257720	EU251403
Agaricomycetes	Agaricales	Marasmioid	Physalacriaceae	*D. tabescens* EU	HAT1S5	HQ232292	HQ285906
Agaricomycetes	Agaricales	Marasmioid	Physalacriaceae	*D. tabescens* EA	CFCC 5906	MG931732	
Agaricomycetes	Agaricales	Marasmioid	Physalacriaceae	*D. tabescens* NA	ATMUS2	AY213588	JF313113
Agaricomycetes	Agaricales	Marasmioid	Physalacriaceae	*A. mellea*-NA	ST20	AY213586	JF313128
Agaricomycetes	Agaricales	Marasmioid	Physalacriaceae	*A. mellea*-NA	D4	HQ232290	HQ285904
Agaricomycetes	Agaricales	Marasmioid	Physalacriaceae	*A. mellea*-EA	CFCC81073	MG931746	

Agaricomycetes	Agaricales	Marasmioid	Physalacriaceae	*A. gemina*	ST9	AY213556	JF313135
Agaricomycetes	Agaricales	Marasmioid	Physalacriaceae	*A. borealis*	A1	AY213552	JF313141
Agaricomycetes	Agaricales	Marasmioid	Physalacriaceae	*A. ostoyae*	C2	JN657459	JN657486
Agaricomycetes	Agaricales	Marasmioid	Physalacriaceae	CBS D	CFCC80932	MG931748	MH002732
Agaricomycetes	Agaricales	Marasmioid	Physalacriaceae	*A. ostoyae*	CFCC83494	MG931722	MH002709
Agaricomycetes	Agaricales	Marasmioid	Physalacriaceae	*A. nabsnona*	M90	AY213573	JF313122
Agaricomycetes	Agaricales	Marasmioid	Physalacriaceae	PS I	NE4	AB510874	AB510771
Agaricomycetes	Agaricales	Marasmioid	Physalacriaceae	PS II Gallica population	ST22	AY213569	JF313126
Agaricomycetes	Agaricales	Marasmioid	Physalacriaceae	PS II Gallica population	NA13	AB510890	AB510760
Agaricomycetes	Agaricales	Marasmioid	Physalacriaceae	PS II Gallica population	HY2a	JN657455	JN657482
Agaricomycetes	Agaricales	Marasmioid	Physalacriaceae	PS II Sin-Cep population	BRNM695717	EU257716	EU251396
Agaricomycetes	Agaricales	Marasmioid	Physalacriaceae	PS II Sin-Cep population	CFCC 80965	MG931712	MH002699
Agaricomycetes	Agaricales	Marasmioid	Physalacriaceae	PS II CSP population	CFCC 88938	MG931755	MH002739
Agaricomycetes	Agaricales	Marasmioid	Physalacriaceae	PS II CSP population	CFCC 88888	MG931737	MH002722
Agaricomycetes	Agaricales	Marasmioid	Physalacriaceae	PS II CSP population	CFCC 84786	MG931731	MH002718
Agaricomycetes	Agaricales	Marasmioid	Schizophyllaceae	*Schzophyllum radiatum*	CBS301_32	LT217537	LT217603
Agaricomycetes	Agaricales	Hygrophoroid	Hygrophoraceae	*Hygrophorus pudorinus*	AFTOL_ID_1723	DQ490631	GU187710
Agaricomycetes	Agaricales	Hygrophoroid	Hygrophoraceae	*Pseudoarmillariella ectypoides*	AFTOL_ID_1557	DQ192175	GU187733
Agaricomycetes	Agaricales	Pluteoid	Amanitaceae	*Amanita modesta*	HKAS75405	KJ466379	KJ481945
Agaricomycetes	Agaricales	Pluteoid	Amanitaceae	*Amanita muscaria*	CMP3143	EU071889	EU071860

Agaricomycetes	Agaricales	Pluteoid	Pluteaceae	*Pluteus romellii*	AFTOL_ID_625	AY854065	AY883433
Agaricomycetes	Agaricales	Plicaturopsidoid	Clavariaceae	*Clavaria zollinge*	AFTOL_ID_563	AY854071	AY881024
Agaricomycetes	Boletales			*Coniophora arida*	MUCL_14244	AM747495	AM747581
Agaricomycetes	Boletales			*Strobilomyces floccopus*	AFTOL_ID 716	AY854068	AY883428
Agaricomycetes	Russulales			*Lactarius deceptivus*	AFTOL_ID_682	AY854089	AY885158
Agaricomycetes	Hymenochaetales			*Fomitiporia mediterranea*	AFTOL_ID_688	AY854080	AY885149
Agaricomycetes	Hymenochaetales			*Fomitopsis pinicola*	AFTOL_ID_770	AY854083	AY885152
Agaricomycetes	Gomphales			*Ramaria rubella*	AFTOL_ID_724	AY854078	AY883435
Dacrymycetes				*Calocera cornea*	AFTOL_ID_438	AY789083	AY881019
Dacrymycetes				*Dacryopinax spathularia*	AFTOL_ID_454	AY854070	AY881020
Tremellomycetes				*Cryptococcus tepidarius*	JCM11965	AB094045	AB920113
