# Supplementary figures and images for: Phylogenetic Relationships, Speciation, and Origin of Armillaria in the Northern Hemisphere: A Lesson Based on rRNA and Elongation Factor 1-Alpha
Source: J Fungi (Basel). 2021 Dec 17;7(12):1088. doi: 10.3390/jof7121088 (PMC8705980; doi:10.3390/jof7121088)

ITS

0.02

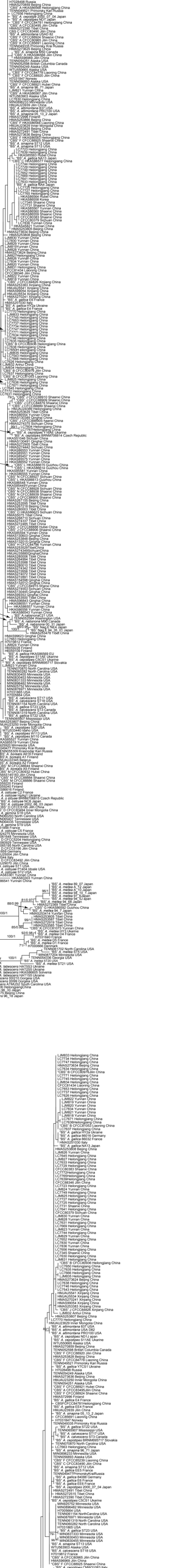

IGS-1

0.04

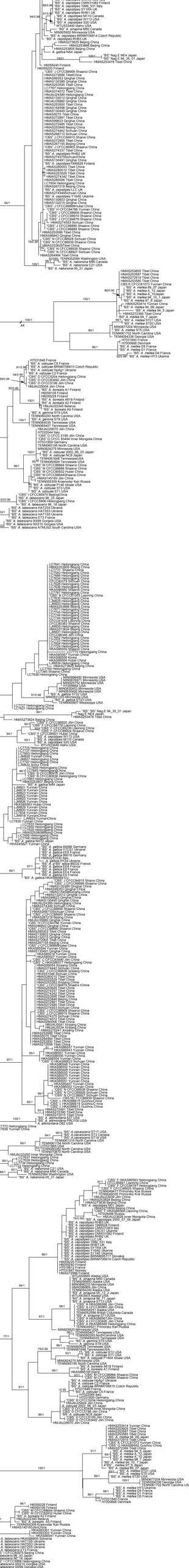

TEF-1α

0.02

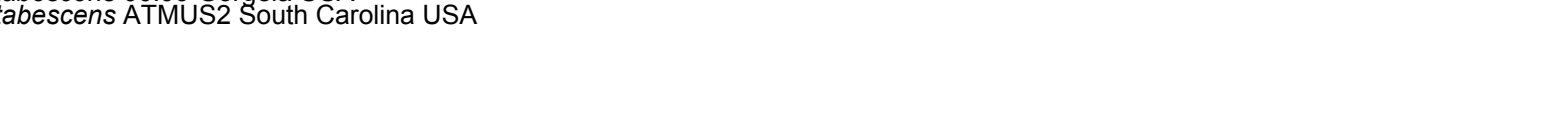

Supplement: Supplementary file 1 [file jof-07-01088-s001.zip › supplementary files/Figure S1 single gene tree.pdf]

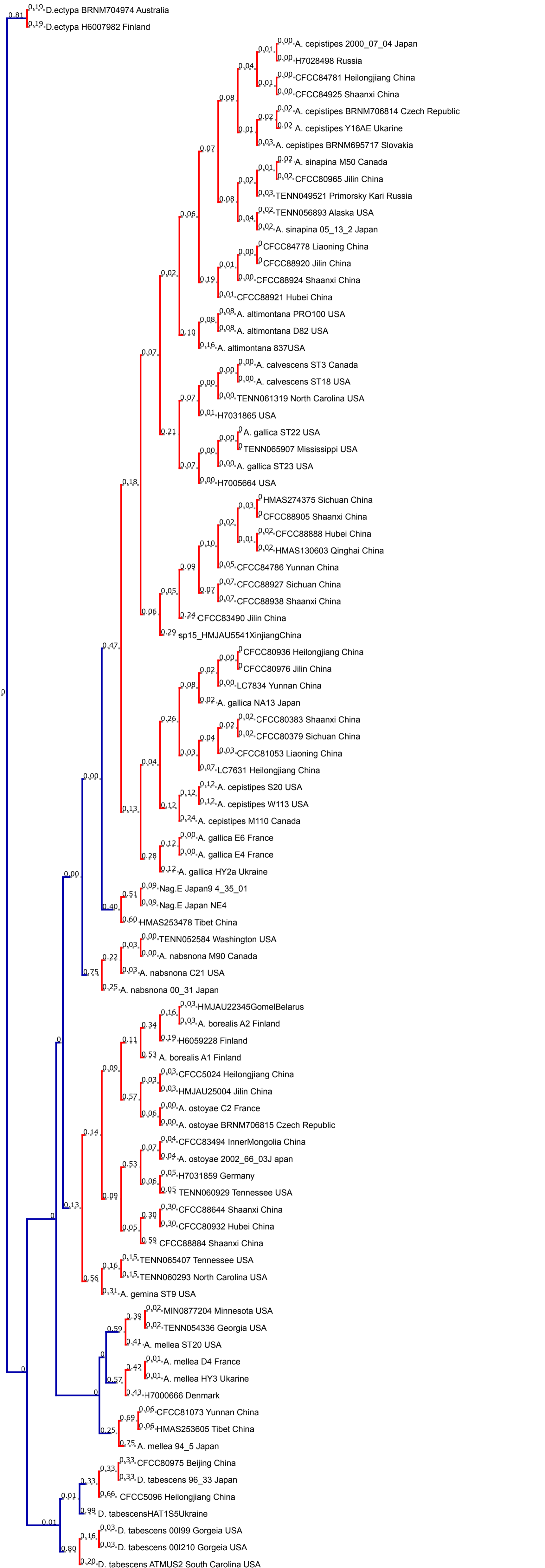

0.10

Supplement: Supplementary file 1 [file jof-07-01088-s001.zip › supplementary files/Figure S2 PTP.pdf]

- ★1 *Quatsinoporites cranhamii*, 125-130 Mya
- ★2 *Archaeomarasmius legetti*, 90 Mya

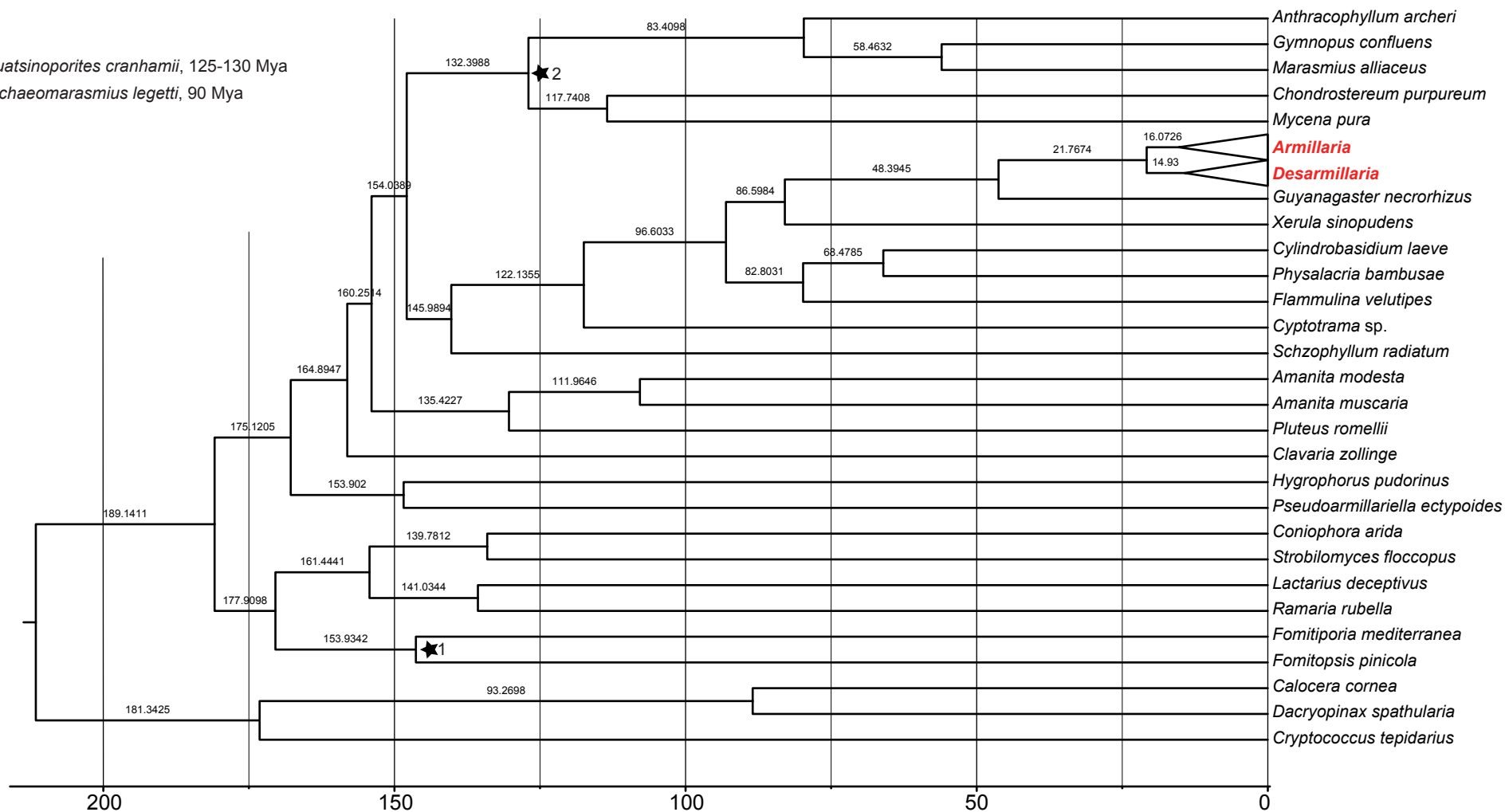

Supplement: Supplementary file 1 [file jof-07-01088-s001.zip › supplementary files/Figure S3 divergence time of armillaria and desarmillaria.pdf]

(a)

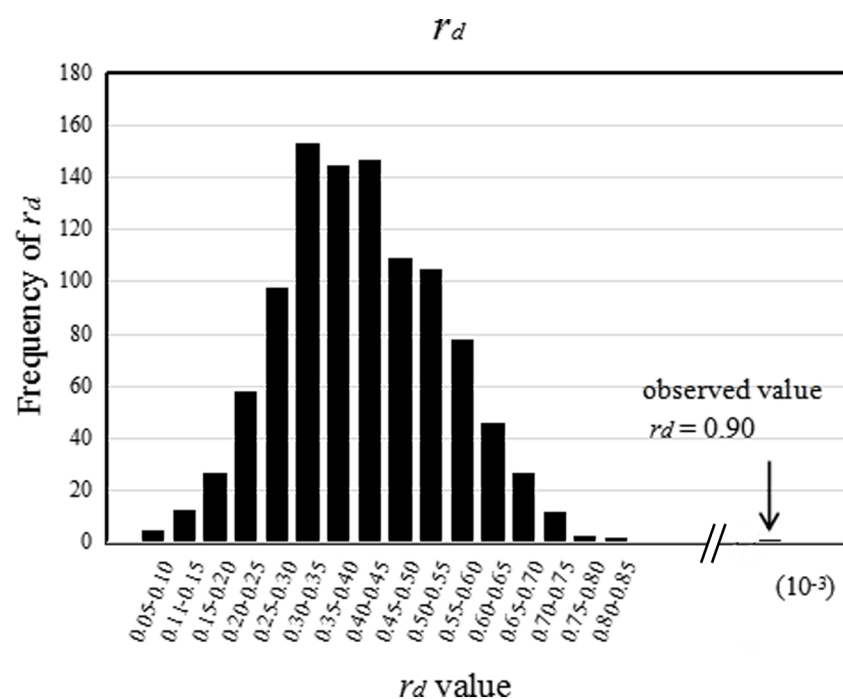

(b)

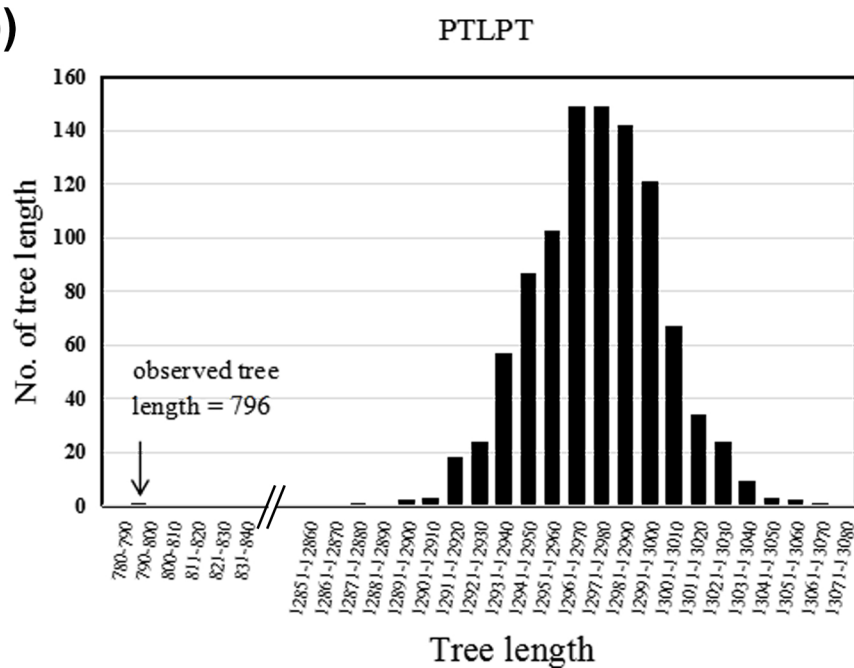

Supplement: Supplementary file 1 [file jof-07-01088-s001.zip › supplementary files/Figure S4 rd and PTLPT.pdf]
